# Performance heuristics for GR(1) synthesis and related algorithms

**DOI:** 10.1007/s00236-019-00351-9

**Published:** 2019-12-05

**Authors:** Elizabeth Firman, Shahar Maoz, Jan Oliver Ringert

**Affiliations:** 1 Tel Aviv University, Tel Aviv, Israel; 2 University of Leicester, Leicester, UK

## Abstract

Reactive synthesis for the GR(1) fragment of LTL has been implemented and studied in many works. In this work we present and evaluate a list of heuristics to potentially reduce running times for GR(1) synthesis and related algorithms. The list includes several heuristics for controlled predecessor computation and BDDs, early detection of fixed-points and unrealizability, fixed-point recycling, and several heuristics for unrealizable core computations. We have implemented the heuristics and integrated them in our synthesis environment Spectra Tools, a set of tools for writing specifications and running synthesis and related analyses. We evaluate the presented heuristics on SYNTECH15, a total of 78 specifications of 6 autonomous Lego robots, on SYNTECH17, a total of 149 specifications of 5 autonomous Lego robots, all written by 3rd year undergraduate computer science students in two project classes we have taught, as well as on benchmarks from the literature. The evaluation investigates not only the potential of the suggested heuristics to improve computation times, but also the difference between existing benchmarks and the robot’s specifications in terms of the effectiveness of the heuristics. Our evaluation shows positive results for the application of all the heuristics together, which get more significant for specifications with slower original running times. It also shows differences in effectiveness when applied to different sets of specifications. Furthermore, a comparison between Spectra, with all the presented heuristics, and two existing tools, RATSY and Slugs, over two well-known benchmarks, shows that Spectra outperforms both on most of the specifications; the larger the specification, the faster Spectra becomes relative to the two other tools.

## Introduction

Reactive synthesis is an automated procedure to obtain a correct-by-construction reactive system from its temporal logic specification [43]. Rather than manually constructing a system and using model checking to verify its compliance with its specification, synthesis offers an approach where a correct implementation of the system is automatically obtained, if such an implementation exists.

GR(1) is a fragment of LTL, which has an efficient symbolic synthesis algorithm [5, 42] and whose expressive power covers most of the well-known LTL specification patterns of Dwyer et al. [15, 32]. GR(1) synthesis has been implemented in several tools in recent years, e.g., RATSY [2] and Slugs [17]. It has been further used and extended in different contexts and for different application domains, including robotics [29], hardware synthesis [3], scenario-based specifications [38], aspect languages [37], event-based behavior models [14], and device drivers [47], to name a few.

In this work we present and investigate performance heuristics for algorithms for GR(1) synthesis in case a specification is realizable and Rabin(1) synthesis [27, 39] in case the specification is unrealizable. For the case of unrealizability we also investigate heuristics for speeding up the computation of unrealizable cores [13, 27], i.e., minimal unrealizable subsets of guarantees that localize a cause of unrealizability. For each heuristics we present (1) its rationale including the source of the heuristics, if one exists, (2) how we implement it on top of the basic algorithms, and (3) a brief intuition about its potential effect.

All heuristics we have developed and studied, satisfy two main criteria. First, they are generic, i.e., they are not optimized for a specific specification or family of specifications. Second, they are conservative, i.e., none of the heuristics changes the results obtained from the algorithms.

We have integrated the heuristics in our synthesis environment Spectra Tools, available together with a set of Eclipse plugins for writing specifications and running synthesis and related analyses, see [53].

We evaluate the presented heuristics on three sets of specifications. The first set, SYNTECH15, consists of 78 specifications of 6 autonomous Lego robots, and the second set, SYNTECH17, a total of 149 specifications of 5 autonomous Lego robots, both written by 3rd year undergraduate computer science students in two project classes we have taught. The third set consists of specifications for the ARM AMBA AHB Arbiter (AMBA) [3] and a Generalized Buffer from an IBM tutorial (GenBuf) [4], which are the most popular GR(1) examples in literature, used, e.g., in [5, 10, 13, 27, 30, 48]. Our evaluation addresses the effectiveness of each of the heuristics individually and together, and whether there exists a difference in effectiveness with regard to different sets of specifications and with regard to the original running time. It further compares our implementation, employing all heuristics, against previously published GR(1) synthesis tools, RATSY [2] and Slugs [17]. To the best of our knowledge, a comprehensive list of heuristics for GR(1) and its systematic evaluation have not yet been published.

A preliminary version of the present work has appeared in [19]. The present work extends this preliminary version by (1) presenting additional heuristics, at the level of predecessor computations and binary decision diagrams, and by (2) adding another set of specifications for evaluation, SYNTECH17, which includes larger and more extensive specifications than SYNTECH15. Further, (3) it provides additional evaluation of all heuristics based on a dissection according to original running times, and finally, (4) reports on a direct comparison of our implementation, with all heuristics employed, against two previously published GR(1) synthesis tools.

The remainder of this work is structured as follows. Section 2 presents required preliminary background on GR(1) synthesis and related algorithms, $\mu$-calculus and fixed-points, unrealizability, and delta debugging. Section 3 presents the three sets of performance heuristics followed by Sect. 4, which presents the evaluation and a discussion of the results. Finally, Sect. 5 presents related work and Sect. 6 concludes.

## Preliminaries

We breifly provide necessary background on LTL and synthesis, $\mu$-calculus and fixed-points, GR(1) synthesis, unrealizability and Rabin(1) game, binary decision diagrams and the computation of controlled predecessors, and delta debugging.

### LTL and synthesis

We repeat some of the standard definitions of linear temporal logic (LTL), e.g., as found in [5], a modal temporal logic with modalities referring to time. LTL allows engineers to express properties of computations of reactive systems. The syntax of LTL formulas is typically defined over a set of atomic propositions $AP$ with the future temporal operators **X** (next) and **U** (until).

The syntax of LTL formulas over $AP$ is $\varphi \,{::}\,=~p~|~\lnot \varphi ~|~ \varphi \vee \varphi ~|~{{{\mathbf {\mathtt{{X}}}}}} \varphi ~|~\varphi {{{\mathbf {\mathtt{{U}}}}}} \varphi$ for $p \in AP$. The semantics of LTL formulas is defined over computations. For $\Sigma = 2^ AP$, a computation $u=u_0u_1..\in \Sigma ^\omega$ is a sequence where $u_i$ is the set of atomic propositions that hold at the *i*th position. For position *i* we use $u,i\models \varphi$ to denote that $\varphi$ holds at position *i*, inductively defined as:

- $$u,i\models p$$ iff $$p \in u_i$$;
- $$u,i\models \lnot \phi $$ iff $$u, i \not \models \phi $$;
- $$u,i\models \varphi _1 \vee \varphi _2$$ iff $$u,i\models \varphi _1$$ or $$u,i\models \varphi _2$$;
- $$u,i\models {{{\mathbf {\mathtt{{X}}}}}} \varphi $$ iff $$u, i{+}1\models \varphi $$;
- $$u,i\models \varphi _1{{{\mathbf {\mathtt{{U}}}}}} \varphi _2$$ iff $$\exists k\ge i{:~} u,k\models \varphi _2$$ and $$\forall j, i\le j < k{:~} u,j\models \varphi _1.$$

We denote $u,0\models \varphi$ by $u \models \varphi$. We use additional LTL operators **F** (finally), **G** (globally), and **H** (historically, i.e., always in the past) defined as:

- $${{{\mathbf {\mathtt{{F}}}}}} \varphi := {\small {{{{\mathbf {\mathtt{{true}}}}}}}} ~{{{\mathbf {\mathtt{{U}}}}}} ~\varphi $$;
- $${{{\mathbf {\mathtt{{G}}}}}} \varphi := \lnot {{{\mathbf {\mathtt{{F}}}}}} \lnot \varphi $$;
- $$u,i\models {{{\mathbf {\mathtt{{H}}}}}} \varphi $$ iff $$\forall 0 \le k \le i{:~} u,k\models \varphi $$.

LTL formulas can be used as specifications of reactive systems, where atomic propositions are partitioned and interpreted as either environment (input) or system (output) variables. An assignment to all variables is called a state.

A winning strategy for an LTL specification $\varphi$ and a set of initial states *I*, prescribes the outputs of a system for all environment choices, such that all computations from *I* will satisfy $\varphi$. The system wins on *I* if there is a winning strategy on *I*. The winning states are all states *W* such that the system wins on *W*.

A specification $\varphi$ is called realizable if a strategy exists such that for all initial environment choices the initial states are winning states. The goal of LTL synthesis is, given an LTL specification, to find a strategy that realizes it, if one exists.

### $\mu$-Calculus and fixed-points

The modal $\mu$-calculus is a fixed-point logic [28]. It extends modal logic with least ($\mu$) and greatest ($\nu$) fixed points. We use the $\mu$-calculus over the power set lattice of a finite set of states *S*, i.e., the values of fixed-points are subsets of *S*. For monotonic functions $\psi$ over this lattice and by the Knaster–Tarski theorem the fixed-points $\mu X.\psi (X)$ and $\nu Y. \psi (Y)$ are uniquely defined and guaranteed to exist. The fixed-points can be computed iteratively [21] in at most |*S*| iterations due to monotonicity of $\psi$:

- $\mu X.\psi (X)$: From $X_0:=\bot$ and $X_{i+1}:=\psi (X_i)$ obtain $\mu X.\psi (X) := X_{f}$ for $X_{f}=\psi (X_f)$ (note $f \le |S|$)
- $\nu Y.\psi (Y)$: From $Y_0:=\top$ and $Y_{i+1}:=\psi (Y_i)$ obtain $\nu Y.\psi (Y) := Y_{f}$ for $Y_{f}=\psi (Y_f)$ (note $f \le |S|$)

The number of iterations of the fixed-point computation is linear in |*S*|. When states are represented by a set of atomic propositions (or Boolean variables) *AP* then $|S|=2^{|AP|}$, i.e., the number of iterations is exponential in *AP*. Because the least (greatest) fixed-point is unique and $\psi$ is monotonic we can safely start the iteration from under-approximations (over-approximations). Good approximations can reduce the number of iterations to reach the fixed-point.

### GR(1) synthesis

GR(1) synthesis [5] handles a fragment of LTL where specifications contain initial assumptions and guarantees over initial states, safety assumptions and guarantees relating the current and next state, and justice assumptions and guarantees requiring that an assertion holds infinitely many times during a computation. The GR(1) realizability problem asks to check whether a winning strategy for the system exists. The GR(1) synthesis problem is to construct a winning strategy, if one exists.

A GR(1) synthesis problem consists of the following elements [5]:

- $\mathcal {X}$ input variables controlled by the environment;
- $\mathcal {Y}$ output variables controlled by the system;
- $\theta ^e$ assertion over $\mathcal {X}$ characterizing initial environment states;
- $\theta ^s$ assertion over $\mathcal {X} \cup \mathcal {Y}$ characterizing initial system states;
- $\rho ^e(\mathcal {X} \cup \mathcal {Y}, \mathcal {X})$ transition relation of the environment;
- $\rho ^s(\mathcal {X} \cup \mathcal {Y}, \mathcal {X} \cup \mathcal {Y})$ transition relation of the system;
- $J^e_{i \in 1..n}$ assertions over $\mathcal {X} \cup \mathcal {Y}$ for the environment to satisfy infinitely often (called justice assumptions);
- $J^s_{j \in 1..m}$ assertions over $\mathcal {X} \cup \mathcal {Y}$ for the system to satisfy infinitely often (called justice guarantees).

Note that realizing (satisfying) a justice assumption (guarantee) $J^e_i$ ($J^s_j$) means realizing (satisfying) the LTL formula **GF**$J^e_i$ (**GF**$J^s_j$).

GR(1) synthesis has the following notion of (strict) realizability [5] defined by the LTL formula^1^:

$$\begin{aligned} \varphi ^{sr}= & {} (\theta ^e \rightarrow \theta ^s) \wedge (\theta ^e \rightarrow {{{\mathbf {\mathtt{{G}}}}}} (({{{\mathbf {\mathtt{{H}}}}}} \rho ^e)\rightarrow \rho ^s))\\&\wedge \left( \theta ^e \wedge {{{\mathbf {\mathtt{{G}}}}}} \rho ^e \rightarrow \left( \bigwedge _{i \in 1..n} {{{\mathbf {\mathtt{{GF}}}}}} J_i^e \rightarrow \bigwedge _{j \in 1..m} {{{\mathbf {\mathtt{{GF}}}}}} J_j^s\right) \right) . \end{aligned}$$

Specifications for GR(1) synthesis have to be expressible in the above structure and thus do not cover the complete LTL. Efficient symbolic algorithms for GR(1) realizability checking and strategy synthesis for $\varphi ^{sr}$ have been presented in [5, 42]. The algorithm of Piterman et al. [42] computes winning states for the system, i.e., states from which the system can ensure satisfaction of $\varphi ^{sr}$. We denote the states from which the system can force the environment to visit a state in *R* by  , also called the controlled predecessors, defined as:1The system winning states are given by the following formula using $\mu$-calculus notation:2The algorithm from [5] for computing the set $W_{sys}$ is shown in Algorithm 1. Note that this algorithm already contains some performance improvements over the naive evaluation of Eq. (2), e.g., the nested fixed-points *Y* are not computed independently for each $J^s_j$ and *Z*; instead the value of *Z* is updated before computing $J^s_{j+1}$. Algorithm 1 stores intermediate computation results in arrays Z[] (line 19), Y[][] (line 16), and X[][][] (line 14). This memory is used for strategy construction [5].

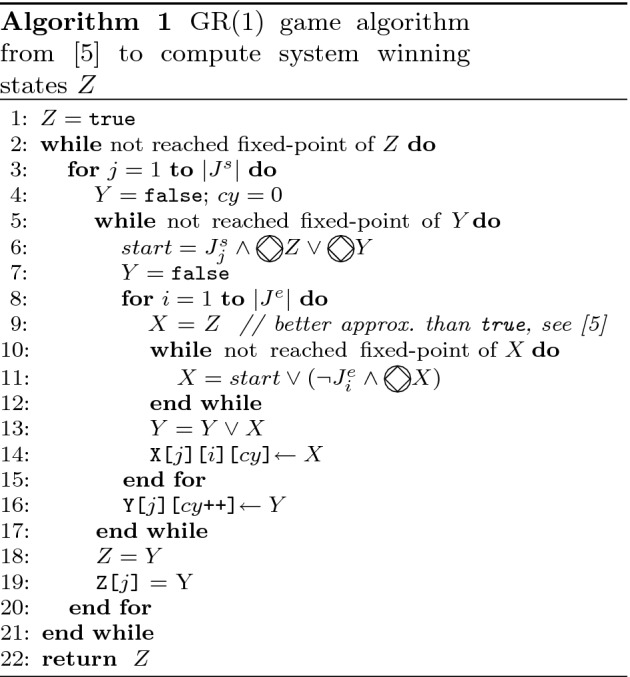



### Unrealizability and Rabin(1) game

A specification $\varphi$ is unrealizable if there is a counter-strategy in which the environment can force the system to violate at least one of its guarantees while satisfying all the environment assumptions. Maoz and Sa’ar [39] show an algorithm for solving a generalized Rabin game with one acceptance pair (Rabin(1) game^2^). The algorithm computes the set of the winning states for the environment by computing cycles violating at least one justice guarantee $J^s_i$ while satisfying all justice assumptions $J^e_j$. Cycles can be left by the system iff the environment can force it to a future cycle (ensures termination) or to a safety guarantee violation.

We denote the states from which the environment can force the system to visit a state in *R* by  defined as:3The set of environment wining states is given by the following formula using $\mu$-calculus notation:4
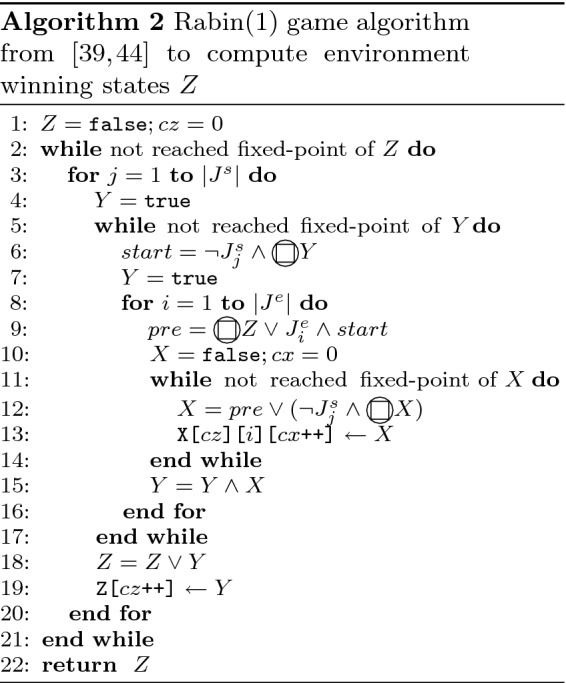


The algorithm from [39] (extended to handle justice assumptions $J^e_i$ as implemented in JTLV [44]) for computing the set $W_{env}$ is shown in Algorithm 2. Again, the algorithm already implements some performance heuristics over the naive implementation of Eq. (4), e.g., the early update of *Z* in line 18. Algorithm 2 stores intermediate computation results in arrays Z[] (line 19) and X[][][] (line 13) for strategy construction.

### BDDs and the computation of controlled predecessors

All the computations and algorithms described above are done symbolically using Boolean functions. The most common way to represent Boolean functions is using Binary Decision Diagrams (BDDs), introduced in [8]. The concept is that for functions representing real life systems, a decision tree, which is exponential in the number of variables, can be reduced to a much smaller directed acyclic graph (DAG), given several rules. The reduction is done by removing duplicate terminals, removing redundant if-then-else tests, and removing duplicate nodes. This sharing of information often results in a compact representation, and when no further optimizations can be applied, the BDD is said to be reduced. Since in a reduced BDD all the nodes are unique, we can define the BDD size as the number of occurring nodes.

Another important rule to adhere to for efficiency is variable ordering, meaning imposing a single occurrence of a Boolean variable along any path in the DAG. Such BDD is called ordered BDD. A reduced ordered BDD that represents a given function is unique, hence we can say that an ordered BDD has a canonical form. All the BDD operations are done on the canonical forms, and binary operations are done on BDDs with compatible variable order. Since the operations may result not in the canonical form of an ordered BDD, the last step is always applying the reduction rules. The reduction on an ordered BDD is essentially a bottom-up DAG traversal, and it takes *O*(*nlog*(*n*)) where *n* is the number of variables. From now on we will refer to the canonical form of an ordered BDD simply as BDD.

We define the following BDD operations:

- not($B_f$): Swaps the *TRUE* and *FALSE* nodes in $B_f$.
- support($B_f$): The support set of the BDD $B_f$, meaning the variables that are used in the BDD representation of function *f*.
- restrict($\{0,1\}$, *x*, $B_f$): Computes the BDD representing a Boolean function *f*[*a* / *x*] for $a\in \{0,1\}$. The computation of restrict(0, *x*, $B_f$) and restrict(1, *x*, $B_f$) is done by redirecting all incoming edges of nodes labeled with *x* to else(x) and then(x) respectively.
- apply(op, $B_f$, $B_g$): A binary operation on Boolean functions *f* and *g*. The resulting BDD represents $f \mathbin {op} g$. This algorithm is based on the Shannon expansion: $f\equiv \bar{x} \cdot f[0/x] + x \cdot f[1/x]$, which is for $f \mathbin {op} g$:
  $$\begin{aligned} f \mathbin {op} g\equiv \bar{x} \cdot (f[0/x] \mathbin {op} g[0/x]) + x \cdot (f[1/x] \mathbin {op} g[1/x]) \end{aligned}$$
  The algorithm is shown in Algorithm 3, and with added caching of intermediate computation the worst time complexity is $O(|B_f||B_g|))$.
- exists(x, $B_f$): Existential abstraction of a Boolean function *f*. Can be implemented as apply(+, restrict(0, *x*, $B_f$), restrict(1, *x*, $B_f$)), but since the two BDDs in the apply are identical down to the level of the *x*-nodes, each *x*-node can be replaced by apply(+, sub-tree of else(*x*)), sub-tree of then(x)). This can be generalized to nested quantifiers in a straightforward way, though the nested problem is NP-complete. We denote this by exists(X, $B_f$) for a set of variables *X*.
- univ(x, $B_f$): Universal abstraction of a Boolean function *f*. Can be implemented as apply($\cdot$, restrict(0, *x*, $B_f$), restrict(1, *x*, $B_f$)), and the same applies as in exists.

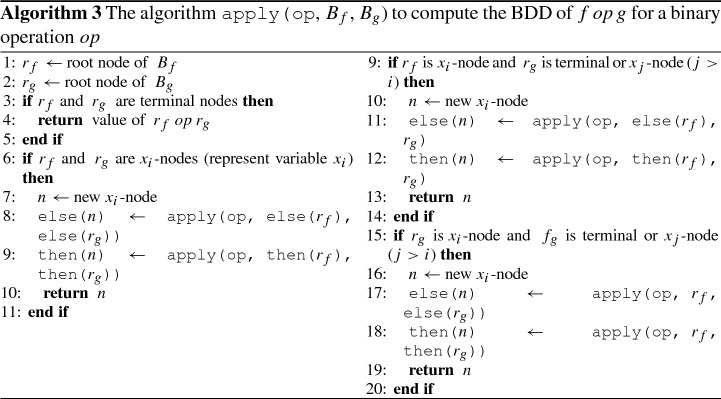


In the GR(1) algorithm, we need to compute  , given in Eq. (1), which is the set of states the system can force the environment to visit. Similarly, in the Rabin(1) algorithm, we need to compute  , given in Eq. (3), which is the set of states the environment can force the system to visit. The computation of this set of states using BDDs and the symbolic representation of states is shown in Algorithms 4 and 5. For the computation we denote the following:


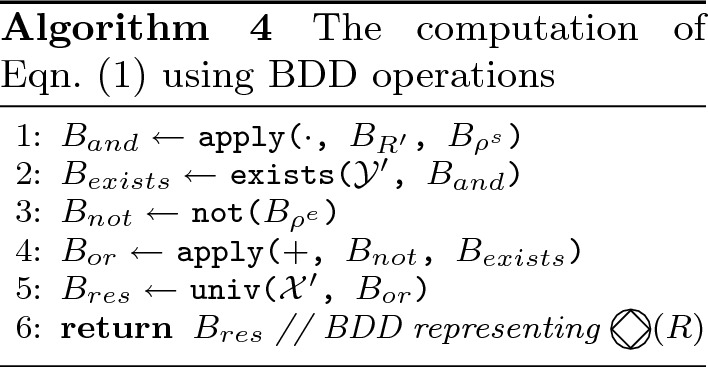

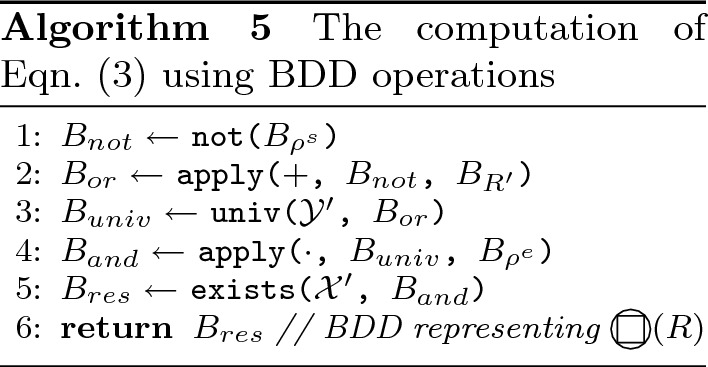

- $B_{R}$ is the BDD of the Boolean function that represents set of states *R*;
- $B_{R'}$ the same BDD as $B_{R}$, but using primed copies of the variables in *R*;
- $B_{\rho ^s}$ is the BDD of the Boolean function that represents the set of the transition relation of the system;
- $B_{\rho ^e}$ as stated above but for the environment;
- $\mathcal {X'}$ the set of the primed copies of the variables in $\mathcal {X}$;
- $\mathcal {Y'}$ as stated above for $\mathcal {Y}$.

### Unrealizable core and delta debugging (DDMin)

Given an unrealizable GR(1) specification, an unrealizable core is a locally minimal subset of system guarantees for which the specification is unrealizable [27]. Such a subset of guarantees is helpful for debugging unrealizable specifications. One way to compute an unrealizable core is using DDMin, described next.

The delta debugging algorithm [52] (DDMin) finds a locally minimal subset of a set *E* for a given monotonic criterion check. We show the DDMin algorithm in Algorithm 6. The input of the algorithm consists of a set *E* and the number *n* of parts of *E* to check. The algorithm starts with $n=2$ and refines *E* and *n* in recursive calls according to different cases (line 3, line 8, and line 14). The computation starts by partitioning *E* into *n* subsets and evaluating check on each subset *part* (line 1) and its complement (line 7). If check holds (line 2 or line 7), the search is continued recursively on the subset *part* (or its complement), until *part* (or its complement) has no subsets that satisfy check. If check neither holds on any subset *part* nor on the complements, the algorithm increases the granularity of the partitioning to 2*n* (line 14) and restarts.

To compute an unrealizable core using DDMin, the method check performs a realizability check for the given subset *part* of system guarantees. 
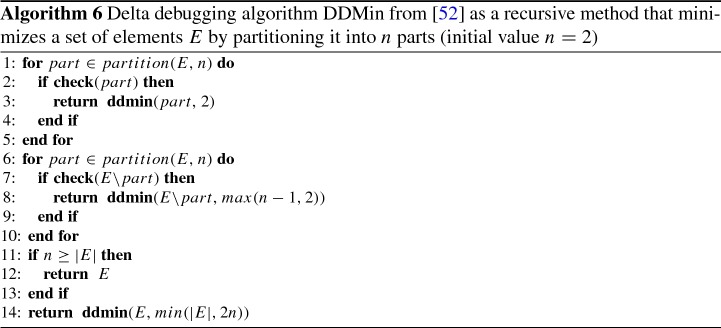


## Suggested performance heuristics

We are now ready to present the main contribution of our work, a list of heuristics for improving running times of GR(1) and related algorithms. We divide the presented heuristics into three lists. The first list is inspired by classic heuristics for the controlled predecessor computation, i.e., the evaluation of the operators  and  (Sect. 3.1). The second list of heuristics applies to the GR(1) and Rabin(1) fixed-point algorithms (Sect. 3.2). Finally, the third list applies to computing unrealizable cores (Sect. 3.3). For each heuristics we present a **rationale** including a source of the heuristics, the **heuristics** and how we implemented it in Algorithms 1–6, and a brief **intution** about its potential effect.

### Controlled predecessor computation and BDDs

#### Grouping of variables and their primed copies

*Rationale* The size of the BDDs depends greatly on the variable order. During the computations the BDDs may grow to the extent that they cannot be computed in a reasonable time if at all, even for small examples. To reduce the BDDs sizes we use dynamic reordering that is supplied by the CUDD package. The CUDD package uses the SIFT algorithm (presented in [46]) for reorder. Roughly, the algorithm iterates over the variables, and moves each of them upwards and downwards the variable order, until eventually choosing the location that results in the smallest BDDs. Note that if the BDDs sizes grow too much while moving a variable in some direction, it stops exploring this course.

In  [40], it was first suggested that instead of moving only individual variables, sifting should move groups of neighboring variables that have strong affinity to each other. Since the main BDD computations are of the controlled predecessors, which relies on the transition relation, the primed and unprimed variables (which are always neighbors) might be strongly related, and as first suggested in [51], it can be beneficial to group them for the dynamic reordering. Since it was first suggested, this heuristics was widely used but was never evaluated on a large set of specifications.

*Heuristics* The CUDD package supplies an API to define groups of neighboring variables. The groups can be defined only during the construction phase and cannot be redefined afterwards. Then, whenever reorder is triggered, it performs the SIFT algorithm, but moves only groups of variables. Note that when variables are moved as a group, they are still moved one by one, and the BDDs sizes change as a result of each swap. The SIFT doesn’t stop if the BDDs grow too much in the intermediate computation, hence it might do more expansive swaps until the whole group is moved. On the other hand, after the whole group is moved the BDDs might get smaller, and an order is found which wouldn’t be found otherwise.

This heuristics is implemented in code executed before Algorithms 1 and 2.

*Intuition* The heuristics is effective when the values of primed and unprimed variables directly influence each other and both would appear in many BDDs together. Then the SIFT algorithm can find better orders more quickly (splitting the variables would increase the number of paths in the BDDs). However, grouping can have negative effects for the SIFT algorithm when no relation between the primed and unprimed copies of a variable exists (these variables still have to be moved around without any benefit).

#### Combined conjunction and existential abstraction

*Rationale* In the computation of both operators,  and  , there is an existential quantification over a disjunction. Since the existential quantification eliminates variables from a BDD, it can save a costly apply on a specific node. The relational product introduced in [9] constructs the existential quantification over a disjunction without ever constructing the disjunction itself, which can be quite large. This algorithm is implemented in the CUDD package, and reported in [23].

*Heuristics* The CUDD package provides an API for using simultaneous conjunction and abstraction (the function Cudd_bddAndAbstract). We use this function when computing the operators in Algorithm 1 line 11 and Algorithm 2 line 12. The computation is described in Algorithm 4 for GR(1) and in Algorithm 5 for Rabin(1), so instead of lines 1 and 2 and lines 4 and 5 respectively, we use the combined function. Note that in Algorithm 5 for Rabin(1) the function is used at the end of the algorithm, whereas in Algorithm 4 for GR(1) the function is used at the beginning, on a potentially much larger BDD. This may affect the effectiveness of the heuristics for the Rabin(1) algorithm compared to the GR(1) algorithm. The function itself implements the relational product from [9]. Its main idea is to perform the ordinary conjunction, but when a node *n* is built labeled with a variable that should be quantified out, in Algorithm 3, instead of returning *n* in line 10, line 13 and line 19 a disjunction is returned, meaning those lines are replaced with **return**apply(+, else(*n*), then(*n*)). Note that this is another recursive step, which causes this algorithm to be exponential in the BDD size.

*Intuition* The heuristics is effective if the conjunction of a BDD with the transition relation creates a large BDD due to primed BDD variables. By quantifying out these variables in the same operation large BDDs are avoided. Depending on the BDD variable order and values, the heuristics might not have any effect. As mentioned above, the worst-case complexity is the same as with separate conjunction and quantification.

#### Partitioned transition relation

*Rationale* Each of the transition relations $\rho ^e$ and $\rho ^s$, are typically represented by a single BDD, which is a conjunction of the respective safety constraints of a specification. The sizes of these BDDs can become very large, and have a negative impact on the computation time of controlled predecessors. Maintaining one large BDD can be costly in general due to reordering. Therefore, as suggested in [9, 20], keeping several smaller transitions and performing the computations with them can be effective in reducing the running time altogether.

An alternative variant of a partitioned transition relation with vector composition was reported in [23]. However, in the context of [23], the input is given as a circuit where it is straightforward to obtain BDDs representing the values of each output variable. Unfortunately, this is not the case for general GR(1) specifications. We have therefore implemented the classic version of partitioning presented in [9] and our algorithm decides the order of quantifications similar to [20].

*Heuristics* Our implementation of this heuristics is based on the iterative computation described in [9] and has two parts, a pre-processing on BDDs of the safety constraints, which is done once, and a revised computation of controlled predecessors, which is repeated on every controlled predecessors computation.

In the pre-processing, instead of building a single transition relation from the safety guarantees, we compute an ordered sequence of transition relations $B_{\rho _{i}^{s}}$ and sets of variables $\mathcal {Y'}_i$: ${(B_{\rho _{1}^{s}}, \mathcal {Y'}_1),\ldots ,{(B_{\rho _{k}^{s}}, \mathcal {Y'}_k)}}$, where $B_{\rho ^s}=B_{\rho _{1}^{s}}\wedge \cdots \wedge B_{\rho _{k}^{s}}$ and $\mathcal {Y'}=\mathcal {Y'}_1\cup \cdots \cup \mathcal {Y'}_k$ is a partition. The same is done for the environment transition using the safety assumptions.

For GR(1), in the computation of the controlled predecessors, described in Algorithm 4, in lines 1 and 2, instead of doing the operations on a single BDD $B_{\rho ^s}$ and quantifying out the whole variable set $\mathcal {Y'}$, we do an iterative computation: starting from $B_{R'}$, each step conjuncts the previous result with the BDD $B_{\rho _{i}^{s}}$, and then quantifies out the set $\mathcal {Y'}_i$ for $0\le i<k$. The same is done for the environment in lines 3–5. The equivalent is done in Algorithm 5 for the Rabin(1) game.

The challenging part is to find the ordered pairs during pre-processing. The order is important because the predecessors computation is correct only if the variables $\mathcal {Y'}_i$ do not appear in the support set of any $B_{\rho _{j}^{s}}$ for all $j>i$ (it is safe to quantify the variables $\mathcal {Y'}_i$ only if they do not appear in subsequent transition relations). It is also important for performance, because in order to reduce the BDD size each iteration of the predecessors computation starts with, we want to quantify out as many variables as possible in each step.

To find an order we start by computing, for each partial transition relation, the set that can be quantified out, which consists of the primed variables that are in the support set of the transition relation. Then we subtract from each set all the other computed sets. The partial transition relation with the maximal set after subtraction, is the one that will be next in the order. If, after subtraction, all sets are empty, we choose the transition with the largest support set, as suggested in [20].

After finding an order, the resulting sets $\mathcal {Y'}_i$ for quantification may be too small to benefit from (there are too many of them). Therefore we create minimal sets of size 3 by conjuncting the partial transitions (sequential in the resulting order) and unifying the sets for the quantification.

*Intuition* This heuristics is effective when safety constraints can be grouped and ordered such that some BDD variables do not appear higher up in the order. By quantifying these variables early, in the predecessor computation the sizes of BDDs will be reduced. In addition, the intermediate quantification operations are on smaller sets of BDD variables and thus faster (quantification has exponential time complexity in the number of quantified BDD variables). The heuristics is less effective if many safety constraints share many variables and do not allow for early quantification.

Examples where the partitioned transition relation might have a negative effect are trivial cases in which the conjunction of safety constraints with the set of target states evaluates to **false**. Here, final quantification would be trivial, but intermediate quantification might take place before the value **false** is determined.

### GR(1) and Rabin(1) fixed-point algorithm

#### Early detection of fixed-point

*Rationale* The GR(1) game and the Rabin(1) game iterate over the justice guarantees in the outermost fixed-point. Each iteration refines the set of winning states based on the justice guarantee and the computed set from the previous iteration (for-loop in Algorithm 1, line 3 and Algorithm 2, line 3). Computing a fixed-point for the same justice guarantee $J^s_j$ and the same set *Z* always yields the same set of winning states. We can exploit the equality to detect if we will reach a fixed-point without completing the for-loop, i.e., without computing the fixed-points for all justice guarantees. We found this heuristics implemented in the Rabin(1) game in JTLV [44]. We have not seen a similar implementation for the GR(1) game.

*Heuristics* For each iteration of the justice guarantees $J^s$ we save the resulting set of winning states for justice $J^s_j$ as Z[*j*] (Rabin(1), Z[*cz*]). Starting in the second iteration of the outermost fixed-point we compare for each justice $J^s_j$ the resulting *Z* of its iteration to the previously computed Z[*j*] (Rabin(1), Z[$cz-|J^s|$]). If the sets are equal the algorithm reached a fixed-point with winning states *Z*. The heuristics is correct since the next iteration of justice $J^s_{j\oplus 1}$ will start from the set Z[*j*] (Rabin(1), Z[$cz-|J^s|$]), which is the same set it started from when it was previously computed. Hence, $\forall k>j:$Z[*k*]=Z[*j*] (Z[$cz-|J^s|$]=Z[$cz-|J^s| + k$]), so by definition we reached a fixed-point for $k=n$ (all justice guarantees).

*Intuition* This heuristics is most effective for GR(1), when in the previous to last iteration of the outer fixed-point only the first justice guarantee changes the set of winning states. Then, the final iteration to confirm all winning states can stop after this first justice guarantee. However, when in the previous to last iteration the last justice guarantee changes the set of winning states, the final iteration has to complete up to that guarantee and the heuristics has no effect. The effect is dual for the Rabin(1) algorithm.

#### Early detection of unrealizability

*Rationale* The GR(1) game and the Rabin(1) game compute all winning states of the system and environment. When running GR(1) synthesis or checking realizability we are interested whether there exists a winning system output for all initial inputs from the environment. When running Rabin(1) synthesis or checking unrealizability we are interested whether there is one initial environment input such that the environment wins for all system outputs. Thus, in both cases it is not necessary to compute all winning states, instead we can stop computation once we can determine the outcome for the initial states.

*Heuristics* The outermost fixed-point in the GR(1) game is a greatest fixed-point. The game starts from the set of all states and refines it to the winning states. Thus, after the computation of the winning states for a justice guarantee we check whether the system still wins from all initial inputs. We implemented this check in Algorithm 1 after line 19. If the system loses for at least one initial environment input we stop the computation of winning states.

The outermost fixed-point in the Rabin(1) game is a least fixed-point. The game starts from an empty set of states and extends it to the winning states. Thus, after the computation of the winning states for a justice guarantee we check whether the environment now wins from some initial input. We implemented this check in Algorithm 2 after line 19. If the environment wins for at least one initial input we stop the computation of winning states.

*Intuition* This heuristics is most effective when the algorithms require many iterations of the outermost fixed-point but initial states are determined to be losing early on. For realizable specifications, this heuristics has no effect.

#### Fixed-point recycling

*Rationale* The GR(1) game and the Rabin(1) game are solved by computing nested fixed-points of monotonic functions [see Eqs. (2) and (4)].

The time complexity of a straightforward implementation of the fixed-point computation is cubic in the state space and can be reduced to quadratic time [7], as mentioned in [5]. This method can also be applied to the Rabin(1) game. Interestingly, although fixed-point recycling is used to obtain quadratic instead of cubic time complexity of the GR(1) algorithm [5] (as measured in number of symbolic operations), to the best of our knowledge, the application of the approach of [7] to GR(1) has never been systematically evaluated.

*Heuristics* Fixed-points are usually computed by fixed-point iteration starting from $\bot$ (least fixed-points) or $\top$ (greatest fixed-points) until a fixed-point is reached. The same principle works for the evaluation of nested fixed-points where for each iteration step of the outer fixed-point, the inner fixed-point is computed from scratch. The main idea of [7] is to exploit the monotonicity of fixed-point computations and start nested fixed-point computations from approximations computed in earlier nested computations. Consider the formula $\mu Z. \nu Y. \mu X.\psi (Z, Y, X)$, iteration $k+1$ of *Z*, and iteration *l* of *Y*: due to monotonicity $Z_k \subseteq Z_{k+1}$ and $Y_{l}^{ of ~Z_{k}} \subseteq Y_{l}^{ of ~Z_{k+1}}$. Thus, the fixed-point *X* for $Z_{k}$ and $Y_{l}^{ of ~Z_{k}}$ is an under-approximation of the fixed-point *X* for $Z_{k+1}$ and $Y_{l}^{ of ~Z_{k+1}}$ (see [7] for more details).

In both, the GR(1) algorithm and the Rabin(1) algorithm, the fixed-point computations also depend on justice assumptions $J^e_i$ and justice guarantees $J^s_j$. This dependence does not interfere with monotonicity of the computation. However, the algorithms compute $|J^e|\cdot |J^s|$ values of the fixed-point *X* for each iteration of *Y* (stored in array X[][][] in Algorithm 1, line 14).

We implemented this heuristics in the GR(1) game Algorithm 1 with a modified start value for the fixed-point computation of *X* in line 9. Unless the algorithm computes the first iteration of *Z* the value of *X* is set to the previously computed result for the same justice assumption $J^e_i$ and justice guarantee $J^s_j$ and same iteration *cy* of *Y*, i.e., *X* is set to memory cell X[*j*][*i*][*cy*] intersected with *Z*. This value is an over-approximation of the greatest fixed-point *X* and its computation likely terminates after fewer iterations.

Similarly, we implemented the fixed-point recycling heuristics in the Rabin(1) game Algorithm 2 with a modified start value for the fixed-point computation of *X* in line 10. Unless the algorithm computes the first iteration of *Z* the value of *X* is set to the previously computed result for the same justice assumption $J^e_i$ and justice guarantee $J^s_j$ for the same iteration of *Y*. This value is an under-approximation of the least fixed-point *X* and its computation likely terminates after fewer iterations. Note that in Algorithm 2 the fixed-point value of *X* is only stored for the last iteration of *Y* (line 13). We had to change the implementation to store *X* for all iterations of *Y* to use fixed-point recycling as described in [7].

*Intuition* This heuristics is most effective with multiple iterations of the outermost fixed-point (recycling starts from the second iteration). The heuristics provides little or no benefit when the numbers of required iterations of the innermost fixed-points are low, i.e., in cases where these can be computed quickly.

It is important to note that this heuristics changes the worst-case running time of both algorithms from $O(|J^e|\cdot |J^s|\cdot |N|^3)$ to $O(|J^e|\cdot |J^s|\cdot |N|^2)$ [5, 7], as measured in number of symbolic operations. However, this comes at the price of additional memory. The increase in number of BDDs kept in memory may make dynamic variable reordering more frequent and more time consuming.

### Unrealizable core computation

#### Contained sets

*Rationale* The delta debugging algorithm DDMin shown in Algorithm 6 might check subsets of guarantees that are contained in previously checked realizable subsets (e.g., after increasing the number of partitions to 2*n* when all other checks failed). In these cases we do not have to execute the costly realizability check: a subset *part* of a realizable set *E* (failure of check(*E*)) is also realizable.

This heuristics was mentioned in [52] and also implemented for unrealizable core computation in [27].

*Heuristics* We extend the generic DDMin algorithm shown in Algorithm 6. Before checking a candidate set $E'$, i.e., executing check($E'$), we look up whether $E'$ is a subset of any previously checked set *E* with negative evaluation of check(*E*).

*Intuition* This heuristics becomes most effective when the checks of the partitions and their complements fail and the granularity of the partitions has to be increased. It would not have any effect if DDMin is recursively executed only on sets where the checks pass, i.e., when finding minimal set is trivial.

#### Incremental GR(1) for similar candidates

*Rationale* Due to the nature of the DDMin algorithm (Algorithm 6), there are multiple calls to check realizability of subsets of guarantees. Some of the subsets share elements. We can try to reuse computation results from previous calls to check for related subsets of guarantees to speed up the computation of fixed-points, both in Rabin(1) and GR(1) games.

*Heuristics* The main idea is to reuse results of previous computations of the GR(1) game (Algorithm 1) or the Rabin(1) game (Algorithm 2). We identified three cases in DDMin (Algorithm 6). In each case we use different methods to reuse the computations from previous rounds.

Case 1: An unrealizable subset *parent* was found (the set *part* in Algorithm 6, line 2) and DDMin descends to perform the search on subsets of *parent*, starting with $n=2$. We examine the differences between *parent* and its current subset of guarantees to check. We have the following scenarios.

1. Only initial guarantees were removed from *parent*: In both the GR(1) and Rabin(1) games we can reuse the winning states (*Z* in Algorithms 1 and 2) that were computed for *parent*, and perform only a simple check of containment of initial states. For GR(1) we check if the system can win from all its initial states. For Rabin(1) we check if the environment can win for at least one of its initial states.
2. Only safety guarantees were removed from *parent*: Since there are fewer constraints the attractors *Y* are larger, hence the set of winning states *Z* can be larger. In GR(1) we compute *Z* using greatest fixed-point, so we cannot reuse the previously computed $Z_{prev}$ to initialize *Z*. However, $Z_{prev}$ is equivalent to the values *Y* stored as Z[*j*] in Algorithm 1, line 19 in the last fixed-point iteration of *Z*. Thus, $Z_{prev}$ is a safe under-approximation of the least fixed-point *Y* and we change the initialization of *Y* in line 4 to $Y=Z_{prev}$.
3. Only justice guarantees were removed from *parent*: We can reuse all information of the previous computation up to the first removed justice guarantee. We reuse the memory $\texttt {Z}_{prev}$, $\texttt {Y}_{prev}$, and $\texttt {X}_{prev}$ from the first iteration of *Z* on *parent* up to the first removed justice guarantee. Then we continue the computation.

Case 2: All subsets *part* of *parent* are realizable and DDMin continues with complements in Algorithm 6, line 7: In this case and for $n>2$ the candidates $E {\setminus } part$ contain previously checked and realizable candidates. Our main observation is that the system winning states for guarantees $E {\setminus } part$ cannot be more than for any of its subsets. We can check realizability of a GR(1) game by initializing its greatest fixed-point *Z* to the intersection of system winning states $Z_{prev}$ of previously computed subsets. Alternatively, we can check realizability with a Rabin(1) game by initializing its least fixed-point *Z* to the union of environment winning states $Z_{prev}$ of previously computed subsets.

Case 3: All subsets and complements are realizable and DDMin increases search granularity in Algorithm 6, line 14: For the new run Case 1 applies (with the previous parent) and Case 2 applies when checking complements of the sets with higher granularity.

*Intuition* As an example, this heuristics is effective when different checked subsets have disjoint winning states and their subsets are checked. Here, Case 2 would immediately detect unrealizability. From the case distinction, it is possible to see that there are executions of the DDMin algorithm where the heuristics does not contribute, e.g., in Case 1 when justice and safety guarantees are removed (this prevents using any of the two initializations).

It is important to note that this heuristics comes at the price of possibly large amounts of additional memory (from every realizability check performed by the DDMin algorithm). The increase in number of BDDs kept in memory may make dynamic variable reordering more frequent and more time consuming.

#### Incremental partitioned transition relation

*Rationale* In Sect. 3.1.3, we presented a partitioning of the transition relation for checking realizability. This heuristics can be applied to every realizability check in the delta debugging algorithm DDMin shown in Algorithm 6. Importantly, instead of computing a good parition on every realizability check (each time on a different subset of the transition relation), we can compute it once and reuse the result.

*Heuristics* The implementation of this heuristics is based on the implementation in Sect. 3.1.3, which has two parts, a pre-processing on BDDs of the safety constraints, and a revised computation of controlled predecessors. Here we break the pre-processing into two phases. In the first phase, which is an actual pre-processing, we try to find the best partitions that can also be reused later in the DDMin algorithm. In the second phase, during each realizability check that is performed for a subset of the guarantees, we finalize the found sets in the pre-processing stage for the given subset of the safety guarantees.

The pre-processing for DDMin has a list of all the support sets of the BDDs of the safety guarantees. Using this list we look for partitions as described in Sect. 3.1.3. We look at the intersection of the sets in the list with the primed variables. Then we search for the disjoint sets by subtracting from each set all the other sets, and choosing the maximal result. If such a set does not exist we take the maximal support set. Thus, we get an ordered mapping from support sets to sets of primed variables to quantify out. Note that the mapping contains only sets of variables and not actual BDDs. In the DDMin algorithm itself, shown in Algorithm 6, each check gets a set of guarantees and uses them to build the transition relation for the realizability check (using GR(1) or Rabin(1) game algorithms). We take these guarantees and build the partitioned transition using the mapping computed during the pre-processing stage. The order and the sets are already computed, but we need to adjust them according to the current subset of guarantees. Finally, we conjunct consecutive sets to have minimal sets of size 3. The revised computation of controlled predecessors is same as described in Sect. 3.1.3.

*Intuition* The heuristics is effective when the DDMin algorithm requires many realizability checks with large numbers of guarantees. Then the partitioning and order of all guarantees computed at the start is reused many times and prevents repeated individual preprocessing for the partitioned transition relation of Sect. 3.1.3. It becomes less effective if the DDMin algorithm does not require many realizability checks.

#### GR(1) game versus Rabin(1) game

*Rationale* GR(1) games and Rabin(1) games are determined: each game is either unrealizable for the system player or unrealizable for the environment player. To check for unrealizability, it is thus equally possible to play the Rabin(1) game or the GR(1) game.

The implementations of Könighofer et al. [27] and Cimatti et al. [13] use the GR(1) game for checking realizability during unrealizable core computation.

*Heuristics* We replace the implementation of check. Instead of playing the GR(1) game we play the Rabin(1) game and negate the result.

*Intuition* Although one algorithm computes exactly the complement set of winning states computed by the other, and the worst-case number of iterations is the same for both, for specific specifications, the number of iterations and/or the computation time of each step may differ.

## Evaluation

We divide the evaluation into three parts following the division of the performance heuristics into heuristics for controlled predecessor computation and BDDs from Sect. 3.1, heuristics for the GR(1) and the Rabin(1) algorithms from Sect. 3.2, and heuristics for computing unrealizable core from Sect. 3.3.

The evaluation aims to address the following three research questions. How effective are the heuristics

1. ...when applied to specifications with different original running times?
2. ...individually and together?
3. ...when applied to specifications from different sets?

Finally, while the main goal of our work is not to outperform existing GR(1) synthesizers but to formally define and empirically investigate a list of suggested heuristics, in order to put our results in context, we present a comparison against two previously presented tools, RATSY [2] and Slugs [17]. Thus, we aim to address a fourth research question:

RQ4 How does the performance of our implementation in Spectra, with all heuristics employed, compare against the performance of previously presented tools?

### Setup

We used the GR(1) game and Rabin(1) game implementations shown in Algorithm 1 and Algorithm 2 as reference (recall that these algorithms already contain performance improvements over naive implementations following the fixed-point formulation, see Sect. 2). We have implemented these two algorithms in C using CUDD 3.0 [49]. We also implemented the heuristics for these algorithms in C, for some using their direct API implementations in CUDD (as mentioned in Sect. 3). We implemented the pre-processing in Java, including the parsing of the specifications and the construction of BDDs for the assumptions and guarantees. The parts of the heuristics that are performed in the pre-processing are also implemented in Java.

Our implementation starts with the BDD variable order as it appears in the specification. We use the default dynamic variable reordering of CUDD. We immediately deallocate BDDs that are no longer used, so the reordering mechanism (which also clears the unused BDDs) would not consider redundant BDDs in the ordering.

#### Setup for RQ1 to RQ3

For the evaluation of the first three research questions, we implemented a test frame that executes a realizability check or the DDMin algorithm using GR(1) or Rabin algorithms with different heuristics, as specified to the test, and measures the running times in nanoseconds. We executed each test for every specification 50 times (see Sect. 4.6), with an exception for two very slow specifications for which we executed the tests 10 times. The cutoff time of a running test is 24 h (the tests are CPU bound and not memory bound). We aggregated all the runs of each test on a specification as a median. The ratios we report are ratios of medians of each heuristics compared to a base case (original implementations of algorithms as shown in Algorithms 1–6) for the same specification. We performed the experiments on an ordinary desktop computer with Intel Xeon W-2133 processor and 32 GB RAM, running Windows 10.

#### Setup for RQ4

For the evaluation of the fourth research question, we used two previously presented tools for performance comparison, RATSY [2] and Slugs [17]. For each, we used the latest available version: RATSY 2.1.0 64-bit version from its website,^3^ and the latest version of Slugs from Github.^4^ Both tools are for Linux environment only and we set them up and build them as described on their websites. RATSY uses NuSMV [12], which uses an unspecified CUDD version. Spectra and Slugs both use the CUDD 3.0 [49] library for BDD computations.

All three tools are set up to use the default dynamic variable reordering of CUDD. We test realizabilty checking time for each tool. Note that in realizability checking time for Spectra we include the building of the memory required for strategy construction. We use Spectra with the presented heuristics from Sects. 3.1 and 3.2. We use RATSY with its default settings (we could not find explicit instructions to use other specific settings for faster realizability check). We use Slugs with the option –fixedPointRecycling, whose description in the tool’s documentation reads “Realizability checking should typically become faster this way”. We set the max memory for the cache in CUDD to 4 GB. We performed all tests 10 consecutive times and report the median time. The cutoff time of a running test is 4 h. We performed the experiments on a VM of a KVM hypervisor, on host OS Windows 10. The hardware used is Intel Xeon W-2133 processor with 32 GB RAM. The VM runs Ubuntu 18.04.1 LTS, with 10 GB RAM and 6 cores.

### Corpus of specifications

Not many GR(1) specifications are available and these were usually created by authors of synthesis algorithms or extensions thereof.

For the purpose of evaluation, we have collected specifications created by 3rd year undergraduate computer science students in two project classes that we have taught. Over the course of a semester, the students have created specifications for real systems, which they actually built in Lego and run throughout specification development (short videos of the robots built by the students are available 5). We collected all non-identical specifications from the version control system used by the students.

In the project class the students have created the following robots: ColorSort—a robot sorting Lego pieces by color; Elevator—an elevator servicing different floors; Humanoid—a mobile robot of humanoid shape; PCar—a self parking car; Gyro—a robot with self-balancing capabilities; and SelfParkingCar—a second version of a self parking car. We call this set of specifications SYNTECH15. The set consists of 78 specifications of which 61 are realizable and 17 are unrealizable.

We have repeated the same class two years later with an improved set of tools that included many of the analyses described in [13, 30, 32, 34], which enabled the students to write larger and more extensive specifications. The students have created the following robots: AirportShuttle—a shuttle car that picks up passengers and returns to a station for maintenance; ConvoyCars—a robot car driving in a convoy following a line and evading obstacles; Elevator—an elevator with automatic doors and different modes; RobotArm—a robotic arm that moves objects; and SIMPLECar—a self parking car. We call this set of specifications SYNTECH17. The set consists of 149 specifications of which 123 are realizable and 26 are unrealizable.

The SYNTECH15 and SYNTECH17 specifications were *not* created specifically for the evaluation in our work on performance heuristics but as part of the ordinary work of the students in the project class. In total, we have collected 227 ($=78+149$) specifications. We consider these GR(1) specifications to be the most realistic and relevant examples one could find for the purpose of evaluating our work.

We used the SYNTECH15 and SYNTECH17 specifications for the evaluation of the first three research questions.

In addition to the specifications created by the students, we considered the ARM AMBA AHB Arbiter (AMBA) [3] and a Generalized Buffer from an IBM tutorial (GenBuf) [4], which are the most popular GR(1) examples in literature, used, e.g., in [5, 10, 13, 27, 30, 48].

For the evaluation of the first three research questions, we included 5 different sizes of AMBA (1 to 5 masters) and 5 different sizes of GenBuf (5 to 40 requests), each in its original version plus the 3 variants of unrealizability described in [13] (justice assumption removed, justice guarantee added, and safety guarantee added). We have thus run our experiments also on 20 AMBA and 20 GenBuf specifications.

For the evaluation of the fourth research question, we included 10 different sizes of AMBA (1 to 10 masters) and 10 different sizes of GenBuf (5 to 90 requests), each in its original version (as given in [13]) plus the 3 variants of unrealizability described in [13]. We have tested all three tools on 40 AMBA and 40 GenBuf specifications.

For details on the specification sizes see Appendix A. All specifications used in our evaluation, the raw data recorded from all runs, and the program to reproduce our experiments are available from [54].

### Validation of heuristics

#### Intuition

To validate our intuition on the working of the heurisitics and their speedup, for each of the heuristics we have created one example specification where the heuristics is very effective and one example specification where it does not yield an improvement of performance. We run Spectra over these example specifications, with and without the respective heuristics, and validated that the better or worse performance is indeed observed as expected.

While these examples are synthetic, artificial specifications, they provide concrete instances that strengthen our confidence and intution about the working of each of the heuristics. We made all these example specifications available and documented as part of the supporting materials for the paper in [54].

#### Correctness

Our implementation of the different heuristics might have bugs, so to ensure correctness of the code we performed the following validations.

*BDDs count* The BDDs are referenced and de-referenced with each usage. To make sure that we do not have inconsistencies in our data structure we used validations of CUDD (like Cudd_DebugCheck and Cudd_CheckKeys).

*Winning states* The set of winning states that are computed in Algorithms 1–2 for realizable specifications should not be affected by the heuristics. We ensure it by computing the complete set of winning states using the original algorithms and comparing the results to the winning states computed by the modified algorithms employing each of the heuristics from Sect. 3.2 separately. As expected, only for the unrealizable specifications the heuristics for detecting unrealizability early computed fewer winning states.

*Game memory* We need to ensure that the game memory allows for strategy construction (memory is different for fixed-point recycling). For that, we have synthesized strategies from the game memory produced when using our heuristics. We then verified the correctness of the strategies by LTL model checking against the LTL specifications for strict realizability of the original GR(1) and Rabin(1) specifications.

*Controlled predecessors* For the partitioned transition relation from Sects. 3.1.3 and 3.3.3, and for the combined conjunction and existential abstraction from Sect.  3.1.2, we compare each result of  and  (which is called multiple times from Algorithms 1–2), against the result of the original computations with the single transition. Since we get the same set of states, the partitioning and the revised computation of the controlled predecessors are correct.

*Unrealizable core* For the DDMin heuristics from Sect. 3.3, we have compared the resulting core of each heuristics to the original unrealizable core computation. Since the heuristics are not on the DDMin itself but on the check, we expected to always get the same core, as we did. Furthermore, we executed DDMin again on the core, to validate the local minimum.

We run the validation implementation once and did not include it in the total running times reported in the evaluation results. Validation using all the above techniques was successful on all 267 specifications used in this work. This provides evidence for the correctness of the heuristics and their implementations.

**Table 1:** Controlled predecessor computation and BDDs heuristics for GR(1)

Quartile	grp	sca	prt	All
219 specs				
MIN	0.72	0.67	0.53	0.44
$Q_1$	0.91	0.96	1.02	0.96
$Q_2$	0.98	0.98	1.04	1.01
$Q_3$	1.01	1.00	1.11	1.04
MAX	6.09	2.85	3.66	2.46
Original running time $<5$ s				
48 specs				
MIN	0.18	0.18	0.04	0.004
$Q_1$	0.58	0.59	0.44	0.22
$Q_2$	1.07	0.68	0.64	0.40
$Q_3$	1.49	0.78	2.21	0.79
MAX	218.69	3.14	27.81	10.53
Original running time $>5$ s				
35 specs				
MIN	0.27	0.48	0.25	0.11
$Q_1$	0.68	0.59	0.54	0.30
$Q_2$	1.14	0.67	0.67	0.43
$Q_3$	1.47	0.78	2.25	1.09
MAX	218.69	3.14	9.32	10.53
Original running time 5–30 s				
MIN	0.18	0.18	0.04	0.004
$Q_1$	0.28	0.47	0.12	0.02
$Q_2$	0.71	0.68	0.27	0.05
$Q_3$	1.52	0.85	1.34	0.42
MAX	4.21	2.20	27.81	3.72
Original running time $>30$ s				

### Evaluation results: RQ1 to RQ3

We now present aggregated running time data from all runs on all specifications with the different heuristics and their combination. We decided to present for all experiments minimum, maximum, and quartiles of ratios. The ratios are of the different heuristics running times to the original running times of the GR(1) game Algorithm 1, the Rabin(1) game Algorithm 2, and DDMin Algorithm 6. For example, the value 0.68 in Table 1, quartile Q2 and column *sca* means that for 50% of the specifications for which the original GR(1) implementation takes more than 5 s, the *sca* heuristics performs at least 1–0.68 $=$ 32% faster than the original implementation. As another example, in the same table, same row, column *all*, the value 0.40 means that for 50% of the specifications for which the original GR(1) implementation takes more than 5 s, using all three heuristics together improves performance by at least 1–0.4 $=$ 60%, i.e., is at least 2.5 times faster than the original implementation. The lower the value in the table, the better the performance of the heuristics relative to the original. All results are rounded to two decimals (or more if otherwise 0).

**Table 2:** Ratios of the heuristics to the original GR(1) running times for realizable and unrealizable specifications

Quartile	Realizable				Unrealizable			
	grp	sca	prt	All	grp	sca	prt	All
SYNTECH15								
MIN	0.77	0.83	0.83	0.76	0.82	0.97	0.93	0.83
$Q_1$	0.91	0.98	1.05	0.96	0.90	0.97	1.02	1.01
$Q_2$	0.97	0.99	1.11	1.01	0.90	0.98	1.03	1.01
$Q_3$	0.98	1.05	1.13	1.04	0.90	0.98	1.04	1.02
MAX	1.11	1.07	2.46	1.52	0.90	1.00	1.19	1.03
SYNTECH17								
MIN	0.20	0.27	0.04	0.02	0.27	0.53	0.29	0.11
$Q_1$	0.95	0.78	0.83	0.76	0.93	0.89	0.87	0.80
$Q_2$	0.98	0.93	1.00	0.96	0.96	0.96	1.01	0.97
$Q_3$	1.07	0.97	1.02	1.01	1.01	0.99	1.05	1.03
MAX	218.69	2.79	4.17	2.07	4.27	1.05	1.54	1.09
AMBA/GenBuf								
MIN	0.18	0.18	0.23	0.05	0.28	0.35	0.15	0.004
$Q_1$	0.82	0.71	1.12	0.93	0.84	0.69	1.11	0.58
$Q_2$	0.93	0.84	1.67	1.05	0.95	0.92	1.19	0.97
$Q_3$	0.99	0.96	2.22	1.22	0.98	0.96	2.02	1.02
MAX	1.12	1.85	2.95	2.50	4.21	3.14	27.81	10.53

For each set of the heuristics presented in Sects. 3.1–3.3, we present the following two types of sets of tables.

*Original running times dissection* A set of four tables that present a dissection of the ratios of running times of a given algorithm according to the original running times: two tables for specifications that a given algorithm runs for less then 5 s and for more than 5 s, then two tables for specifications that a given algorithm runs in 5 to 30 s, and in more than 30 s. The tables show the ratios of the different heuristics running times to the original running times as explained above. Tables 1 and 3 show the ratios of running times over all 267 realizable and unrealizable specifications. Table 5 shows the ratios of running times over all 73 unrealizable specifications.

*Specification dissection* A set of three tables that present the ratios of running times for heuristics separately for realizable and unrealizable specifications from the sets SYNTECH15, SYNTECH17, and AMBA and GenBuf. The tables show the ratios of the different heuristics running times to the original running times, as explained above. Tables 2 and 4 show the ratios of running times for 61 realizable SYNTECH15 specifications (top left), for 123 realizable SYNTECH17 specifications (middle left), and for 10 realizable AMBA and GenBuf specifications (bottom left), as well as for 17 unrealizable SYNTECH15 specifications (top right), for 26 unrealizable SYNTECH17 specifications (middle right), and for 30 unrealizable AMBA and GenBuf specifications (bottom right). Table 6 shows the ratios of running times for the same unrealizable sets of specifications.

We have run all experiments described above separately for the GR(1) algorithm and for the Rabin(1) algorithm. In almost all experiments, the effect of the heuristics on the performance of the two algorithms was very similar. Thus, to make the presentation concise, below we only report the results for the GR(1) algorithm and mention the results for the Rabin(1) algorithm in footnotes, only in a few cases where they are indeed different. The complete results for the Rabin(1) algorithm are available together with all other results in [54].

In the remainder of this section, Sect. 4.4.1 presents results for controlled predecessor computation and BDDs (from Sect. 3.1), 4.4.2 presents results for GR(1)/Rabin(1) fixed-point algorithms (from Sect. 3.2), 4.4.3 presents results for unrealizable core computation (from Sect. 3.3). For each of these, we discuss the three RQs. Finally, Sect. 4.4.4 presents a summary of answers to the RQs.

#### Results for controlled predecessor computation and BDDs

We present the ratios of running times for heuristics from Sect. 3.1, where the different heuristics are abbreviated as follows: *grp* is grouping of variables and their primed copies from Sect. 3.1.1, *sca* is the combined conjunction and existential abstraction from Sect. 3.1.2, and *prt* is the partitioned transition relation from Sect. 3.1.3. By *all* we refer to the use of all these heuristics together.

*RQ1: Effectiveness of heuristics with regard to different original running times* For the GR(1) algorithm, for 82% of the specifications, realizability checking completes in less than 5 s, and for these (Table 1) we see little to no effect.

For specifications that take more than 5 s (Table 1), we see a greater effect. The grouping of primed and unprimed variables (Sect. 3.1.1) is better for 25% of the specifications by at least 42% (column *grp*). On the other hand, it has a negative effect on 50% of the specifiacations. The combined conjunction and existential abstraction (Sect. 3.1.2) improves running times of 50% of the specification by at least 32% (column *sca*). The partitioning of the transition relation (Sect. 3.1.3) improves running times of 50% of the specifictions by at least 36%, and the use of all the heuristics combined (column *all*) improves running times by at least 60%.

Moreover, the partitioning of the transition relation is typically more effective as the original running time increases. For 50% of the specifications that originally run for 5 to 30 s (Table 1), the improvement is by at least 33%, and for specifications that originally run for more than 30 s (Table 1) the improvement is by at least 73%. The grouping of primed and unprimed variables (Sect. 3.1.1) also seems to be more effective for higher original running times. For 25% of the specifications that originally run for 5 to 30 s, the improvement is by at least 32%. This increases to an improvement of at least 69% for 50% of the specifications that originally run for more than 30 s. Finally, we can also see that the negative effect decreases, i.e., for 50% of the specifications that originally run for 5 to 30 s, the grouping is worse by at least 1.14 times than the original implementation, while for specifications that originally run for more than 30 s the ratio is still smaller than 1 for 50% of the specifications. More generally, almost in all quartiles the ratio of running times between the grouping heuristics and the original implementation is smaller for the specifications that originally run for more than 30 s than for ones that originally run for 5 to 30 s.

*RQ2: Effectiveness of heuristics individually and together* The heuristics of grouping variables and their primed copies reduces running times by at least 5% on 25% of both realizable and unrealizable specifications for the GR(1) algorithm (Table 2, *grp*). Also, for this heuristics we see the worst maximal running time ratio.

The combined conjunction and existential abstraction (Sect. 3.1.2), when applied to the GR(1) algorithm, reduces running times of 25% of the realizable and unrealizable specifications by at most 29%. We can also see in Table 1 (column *sca*) that it performs better for 75% of the specifications by at least 22%.^6^

The heuristics of the partitioned transition relation appears ineffective for most realizable and unrealizable specifications (column *prt*). This might be due to the overhead described in Sect. 3.1.3 and the original low running times, since as described above in RQ1, for specifications with original running time greater than 5 s, the running times of 50% of the specifictions improves by at least 36%.^7^

*RQ3: Difference between specification sets* The SYNTECH17 specifications are the only ones for which we see a positive effect with the partitioned transition relation heuristics (column *prt*). For 25% of both realizable and unrealizable specifications, we see 13–17% improvement in running times. This can be attributed to the nature of the specifications as described in Sect. 4.2, which have larger and more complicated set of guarantees.

For AMBA and GenBuf we get the greatest improvement in running times for 25% of the unrealizable specifications. For all of the heuristics combined (columns *all*) the running times decreases by at least 42%. This might be due to this set of specifications being the largest unrealizable set, which also contains the largest percentage of large (and slow) specifications. In comparison, for the set of SYNTECH15 specifications we observe almost no improvement, and this is the set of the smallest (and fastest) specifications.

#### Results for GR(1)/Rabin(1) fixed-point algorithms

We present the ratios of running times for heuristics from Sect. 3.2, where the different heuristics are abbreviated as follows: *efp* is the early fixed-point detection from Sect. 3.2.1, *eun* is the early unrealizability detection from Sect. 3.2.2, and *fpr* is the fixed-point recycling from Sect. 3.2.3. By *all* we refer to the use of all these heuristics together.

**Table 3:** Fixed-point heuristics for GR(1)

Quartile	efp	eun	fpr	All
219 specs				
MIN	0.61	0.01	0.60	0.01
$Q_1$	0.98	0.99	0.95	0.91
$Q_2$	1.00	1.00	1.00	0.98
$Q_3$	1.00	1.00	1.00	1.00
MAX	1.13	1.11	1.15	1.12
Original running time $<5$ s				
48 specs				
MIN	0.41	0.001	0.42	0.001
$Q_1$	0.87	0.87	0.87	0.70
$Q_2$	0.96	1.00	0.94	0.84
$Q_3$	1.00	1.00	1.00	0.92
MAX	1.33	1.21	1.17	1.07
Original running time $>5$ s				
35 specs				
MIN	0.76	0.01	0.73	0.01
$Q_1$	0.87	0.90	0.86	0.72
$Q_2$	0.94	1.00	0.95	0.83
$Q_3$	0.99	1.00	1.00	0.92
MAX	1.08	1.21	1.17	1.05
Original running time 5–30 s				
13 specs				
MIN	0.41	0.001	0.42	0.001
$Q_1$	0.88	0.42	0.88	0.41
$Q_2$	1.00	1.00	0.94	0.86
$Q_3$	1.00	1.00	1.00	0.94
MAX	1.33	1.07	1.03	1.07
Original running time $>30$ s				

*RQ1: Effectiveness of heuristics with regard to different original running times* The GR(1) fixed-point algorithm (Sect. 3.2) have some effect on specifications for which it runs in less than 5 s (Table 3). For 25% of the specifications, all the heuristics combined (column *all*) are faster by at least 9%. However, for 25% of the specifications that take longer than 5 s to complete, all the heuristics combined improve the running time by at least 30%, and for 75% of these specifications we see an improvement by at least 8% in running time.

For specifications whose original running time is greater than 5 s, most of the heuristics have similar effect, regardless of the increasing original running times. For early detection of unrealizability (column *eun*), for 25% of the specifications that originally take more than 30 s, the running times improve by at least 58% for, while for 25% of the specifications that take 5 to 30 s the improvement is only by at least 10%. This result is a bit misleading since most of the specifications that originally take more than 30 s are unrealizable, so it entails that early unrealizability detection will perform better for this case.

Finally, these heuristics are much more conservative than the previous ones (Sect. 3.1), as we can see from the maximal ratios.

**Table 4:** Ratios of the heuristics to the original GR(1) running times for realizable and unrealizable specifications

Quartile	Realizable				Unrealizable			
	efp	eun	fpr	All	efp	eun	fpr	All
SYNTECH15								
MIN	0.61	0.94	0.6	0.53	0.94	0.36	0.87	0.36
$Q_1$	0.95	1.00	0.93	0.90	0.98	0.73	0.97	0.74
$Q_2$	0.99	1.00	0.96	0.95	1.00	0.88	0.99	0.88
$Q_3$	1.00	1.02	1.00	0.98	1.02	0.91	1.01	0.91
MAX	1.09	1.11	1.10	1.12	1.13	0.95	1.15	0.96
SYNTECH17								
MIN	0.62	0.76	0.66	0.47	0.88	0.33	0.89	0.33
$Q_1$	0.84	1.00	0.84	0.77	0.98	0.88	0.99	0.88
$Q_2$	0.95	1.00	0.95	0.91	0.99	0.92	1.00	0.93
$Q_3$	1.00	1.00	0.99	0.98	1.00	0.97	1.00	0.97
MAX	1.03	1.19	1.05	1.37	1.01	1.00	1.49	1.42
AMBA/GenBuf								
MIN	0.83	0.97	0.74	0.66	0.85	0.001	0.93	0.001
$Q_1$	0.93	0.99	0.83	0.82	0.99	0.10	0.99	0.10
$Q_2$	0.99	1.00	0.92	0.90	1.00	0.54	1.00	0.52
$Q_3$	1.00	1.00	0.95	0.94	1.00	0.97	1.02	0.97
MAX	1.00	1.01	0.96	0.96	1.33	1.07	1.06	1.07

*RQ2: Effectiveness of heuristics individually and together* The heuristics of early fixed-point detection reduces running times by at least 5% on 25% of the realizable specifications (Table 4, *efp*), but seems even less effective on unrealizable specifications. As expected, the early detection of unrealizability has no notable effect on realizable specifications (Table 4, *eun*), but on unrealizable specifications reduces running times of 50% of the specifications by at least 12%/8%/46%. The heuristics of fixed-point recycling appears ineffective for unrealizable specifications, but reduces running times of 25% of the realizable specifications by at least 7%/16%/17% (Table 4, *fpr*). As good news, the combination of all heuristics typically improves over each heuristics separately (column *all*).^8^

*RQ3: Difference between specification sets* For realizable specifications, we see that the suggested heuristics perform better on the AMBA and GenBuf set and SYNTECH17 set than on SYNTECH15, i.e., for GR(1) algorithm all heuristics (columns *all*) decreases running times on 50% of the AMBA and GenBuf and SYNTECH17 specifications by at least 9%, and for SYNTECH15 specifications by at least 5%. A more significant difference between the specification sets is observed for unrealizable specifications. Here the speedup for 50% of the specifications, mainly obtained by *eun*, is at least around 10% for SYNTECH15 and around 7% for SYNTECH17 but at least around 50% for AMBA and GenBuf. We believe that this difference is due to the systematic and synthetic reasons for unrealizability added by Cimatti et al. [13].

#### Results for unrealizable core computation

We present the ratios of running times for heuristics from Sect. 3.3 for unrealizable specifications. The different heuristics are abbreviated as follows: *opt1* is all the heuristics from Sects. 3.1 and 3.2 combined, *opt2* is the heuristics from *opt1* with the heuristics for partitioned transition for DDMin from Sect. 3.3.3 replacing the heuristics from Sect. 3.1.3, *sets* is the contained sets in the core computation from Sect. 3.3.1, and *inc* is the incremental algorithm for similar candidates from Sect. 3.3.2. Here, by *all* we refer to the combination of *sets* and *opt2*, since *opt1* is contained in *opt2* and *inc* does not seem to improve running times.

**Table 5:** DDMin heuristics for GR(1)

Quartile	opt1	opt2	sets	inc	All
52 specs					
MIN	0.39	0.38	0.68	0.66	0.38
$Q_1$	0.96	0.95	0.95	0.99	0.91
$Q_2$	1.03	1.02	0.98	1.01	0.98
$Q_3$	1.06	1.04	0.99	1.03	1.02
MAX	1.44	1.74	1.00	1.10	1.69
Original running time $<5$ s					
21 specs					
MIN	0.03	0.02	0.59	0.71	0.01
$Q_1$	0.15	0.14	0.69	0.93	0.06
$Q_2$	0.30	0.29	0.93	1.00	0.23
$Q_3$	0.34	0.42	0.98	1.07	0.33
MAX	1.00	1.26	1.07	1.42	1.18
Original running time $>5$ s					
12 specs					
MIN	0.17	0.11	0.59	0.71	0.10
$Q_1$	0.30	0.28	0.64	0.91	0.22
$Q_2$	0.33	0.35	0.76	0.98	0.28
$Q_3$	0.46	0.50	0.94	1.05	0.37
MAX	1.00	1.26	1.00	1.42	1.18
Original running time 5–30 s					
9 specs					
MIN	0.03	0.02	0.88	0.78	0.01
$Q_1$	0.08	0.05	0.94	1.00	0.02
$Q_2$	0.14	0.14	0.96	1.00	0.06
$Q_3$	0.20	0.20	1.00	1.10	0.21
MAX	0.32	0.43	1.07	1.41	0.43
Original running time $>30$ s					

*RQ1: Effectiveness of heuristics with regard to different original running times* The core computation heuristics (Sect. 3.3) have some effect on specifications that are faster than 5 s (Table 5). For 25% of the specifications all the heuristics combined (column *all*) are faster by at least 9%. There is a notable effect on specifications that are slower than 30 s. For all the specifications the DDMin is faster by at least 57%. There are few specifications for which the DDMin did not complete until the cutoff time (24 h), and for these we count the original running time as 24 h. For this reason we have ratios that show improvement of 99%.

All the heuristics combined from Sects. 3.1–3.2 are more effective as the original running times increase (column *opt1*). For specifications that run originally for 5 to 30 s, the running time is faster by at least 67% for 50% of the specifications, and for all the specifications that run longer than 30 s the running time is better by at least 68%.

The contained sets heuristics (Sect. 3.3.1) shows less effect as the original running time grows, for specifications that run more than 5 s (column *sets*). It is most effective for specifications with original running time from 5 to 30 s. For 50% of the specifications there is an improvement of 24%. This heuristics is less effective by itself on large specifications since the first iterations run on very large sets, and without any performance heuristics for these expensive realizability checks we get running times close to the original ones. However, we can see an effect in the combination of all the heuristics (column *all*). For 50% of the specifications with original running time greater than 30, the improvement in running times is at least 94%, where without contained sets heuristics (column *opt2*) there is an improvement of at least 86%.

Finally, for the incremental algorithm for similar candidates (Sect. 3.3.2), for 25% of the specifications that originally run for more than 5 s there is an improvement of at least 7%.^9^

**Table 6:** Ratios of the heuristics to the original DDMin running times for unrealizable specifications

Quartile	DDmin with GR(1)				
	opt1	opt2	sets	inc	All
SYNTECH15 unrealizable					
MIN	0.43	0.45	0.59	0.86	0.33
$Q_1$	1.02	0.99	0.96	1.01	0.95
$Q_2$	1.05	1.04	0.98	1.01	1.02
$Q_3$	1.07	1.04	0.99	1.01	1.02
MAX	1.10	1.07	1.00	1.02	1.04
SYNTECH17 unrealizable					
MIN	0.17	0.11	0.59	0.66	0.10
$Q_1$	0.42	0.45	0.93	0.94	0.44
$Q_2$	0.92	0.91	0.98	0.99	0.91
$Q_3$	1.02	1.01	0.99	1.01	0.99
MAX	1.04	1.03	1.00	1.02	1.03
AMBA/GenBuf unrealizable					
MIN	0.03	0.02	0.65	0.93	0.01
$Q_1$	0.28	0.28	0.89	1.03	0.22
$Q_2$	0.78	0.81	0.95	1.05	0.62
$Q_3$	1.03	1.07	0.98	1.07	1.03
MAX	1.44	1.74	1.07	1.42	1.69

*RQ2: Effectiveness of heuristics individually and together* Using the combined heuristics (columns *all*) appears effective on all specifications and reduces running times for 60% of the specifications (considering the number of unrealizable specifications represented by each table in Table 6).

The heuristics of contained sets (column *sets*) has at least 4% improvement for 25% of the specifications.

The heuristics of *opt1* and *opt2* seem to be the most effective for most specifications. The difference between them is that in *opt1* the partitioning of the transition relation is performed in each iteration of Algorithm 6 (in check), but in *opt2* the partitioning is done in the pre-processing (Sect. 3.3.3). We can see that the improvement they both yield is very similar, and sometimes *opt1* is unexpectedly better than *opt2*. For instance, for 50% of the AMBA and GenBuf specifications with the GR(1) implementation (Table 6), the running times are reduced by 22% with *opt1* and by 19% with *opt2*. This means that the partitioning and the order of quantifications that is found in every iteration of DDMin (for different sets) might be less expensive in computation time than computing it once in the pre-processing stage and then searching for the information in every iteration of DDMin.

Contrary to our expectation, the reuse of previous BDDs for incremental game solving does not improve running times for almost all specifications (columns *inc*). It does yield a small improvement for SYNTECH17 of 6% for DDMin.

The largest factors are seen in columns *opt1* and *opt2*. This shows that they are riskier than other heuristics.

*RQ3: Difference between specification sets* Looking at the specifications from top to bottom in Table 6, we see increasing improvement in running times for almost all heuristics (columns *opt1*, *opt2*, *sets*, *all*). This can be attributed to the increasing original running times. Another observation is that the reuse of previous BDDs for incremental game solving is beneficial only for SYNTECH17 specifications. It is interesting since it is the only set of specifications that has large specifications that were not created systematically.

#### Results summary

*RQ1: Effectiveness of heuristics with regard to different original running times* For all the heuristics presented in this work, it is apparent that for specifications where the original running time is greater than 5 s, there is a great improvement. For all the heuristics of controlled predecessor computation and BDDs (Sect. 3.1) combined, we observe an improvement of at least 60% for 50% of the specifications. For all the heuristics of GR(1)/Rabin(1) fixed-point algorithms (from Sect. 3.2) combined, we observe an improvement of at least 15% for 50% of the specifications. For all the heuristics of unrealizable core computation (from Sect. 3.3) combined, we observe an improvement of at least 67% for 75% of the specifications.

*RQ2: Effectiveness of heuristics individually and together* For all the heuristics presented in this work, the best improvements in running times are for all the heuristics combined. Some heuristics perform better on some specifications and have negative effect on others. Yet, the combination of all the heuristics mostly mitigates the negative effects and results in the best running time. The heuristics of controlled predecessor computation and BDDs (Sect. 3.1) are the most volatile. For 15% of the specifications some of the heuristics have negative effect when used individually, but this effect is mostly mitigated by running all of the heuristics combined. The heuristics of GR(1)/Rabin(1) fixed-point algorithms (from Sect. 3.2) are more conservative. They result in more moderate improvements, but typically have no negative effect. Some of the heuristics presented for unrealizable core computation (from Sect. 3.3) proved to be ineffective, but the combination of the heuristics that improve the running times provided the best results.

*RQ3: Difference between specification sets* Two main differences between the specification sets seem to relate to the performance of the heuristics: the size of the specifications and the type of construction (by students or systematically). The SYNTECH15 set typically shows the least improvements for most of the heuristics, and it is the set of the smallest specifications. The SYNTECH17 set has larger specifications and it shows better results than SYNTECH15 for most of the heuristics. Both SYNTECH15 and SYNTECH17 were created by students in a project class for the development of Lego robots (see Sect. 4.2). The AMBA and GenBuf specifications are larger than the SYNTECH15 specifications but not necessarily larger than the SYNTECH17 specifications. However, they were created systematically by researchers, and typically have the best results for the applied heuristics. Still, some heuristics (partitioned transition relation in Sects. 3.1.3 and 3.3.3) are more effective for SYNTECH17 specifications. This may be attributed to their size and unsystematic construction.

### Evaluation results: RQ4

We present a comparison of the running times between the Spectra tool and the RATSY and Slugs tools. We show the results in two sets of tables, one for the AMBA specifications, and the other for the GenBuf specifications. Each set includes 4 tables, one for each specification type: the original realizable version, and the 3 unrealizable versions (see Sect. 4.2). In each table, the first column represents the size of the specification: the number of masters and the number of senders for AMBA and GenBuf respectively. For the Spectra tool we present absolute running times in seconds, while for the RATSY and Slugs tools we present a ratio. The ratio represents Spectra’s running time to RATSY’s and Slugs’ running times. For example, the value 28.05 in Table 7, means that for the realizable AMBA specification with a single master, RATSY was 28 times faster than Spectra. In this case the running time of Spectra was 4.42 s.

**Table 7:** Tool comparison over AMBA specifications

No. of masters	Spectra absolute time	Spectra/RATSY	Spectra/Slugs
1	4.42	28.05	24.82
2	5.14	1.43	2.36
3	12.51	0.19	0.37
4	55.88	0.04	0.08
5	86.50	0.007	${{-}{-}}$
6	150.36	${{-}{-}}$	${{-}{-}}$
7	287.87	${{-}{-}}$	${{-}{-}}$
8	1420.19	${{-}{-}}$	${{-}{-}}$
9	1301.36	${{-}{-}}$	${{-}{-}}$
10	3562.72	${{-}{-}}$	${{-}{-}}$
Realizable specifications			
1	4.36	60.60	73.16
2	4.57	20.07	13.60
3	4.84	2.38	1.72
4	5.64	0.16	0.25
5	6.88	0.02	0.01
6	8.28	0.003	0.002
7	9.25	${{-}{-}}$	${{-}{-}}$
8	17.03	${{-}{-}}$	${{-}{-}}$
9	22.34	${{-}{-}}$	${{-}{-}}$
10	21.24	${{-}{-}}$	${{-}{-}}$
Unrealizable: justice assumption removed			
1	4.38	110.16	54.69
2	4.51	25.77	8.68
3	4.62	10.06	1.19
4	5.53	0.75	0.10
5	6.79	0.02	0.007
6	7.96	0.002	0.002
7	7.19	0.001	${{-}{-}}$
8	13.35	${{-}{-}}$	0.001
9	15.48	${{-}{-}}$	${{-}{-}}$
10	25.60	${{-}{-}}$	${{-}{-}}$
Unrealizable: additional justice guarantee			
1	4.35	114.76	91.81
2	4.49	48.37	11.46
3	4.55	40.21	4.37
4	5.08	4.55	0.25
5	5.49	0.02	0.006
6	6.59	${{-}{-}}$	${{-}{-}}$
7	7.14	0.01	0.0008
8	8.71	$\times \times$	${{-}{-}}$
9	12.47	$\times \times$	${{-}{-}}$
10	16.57	$\times \times$	${{-}{-}}$
Unrealizable: additional safety guarantee			

Since for some specifications RATSY and Slugs were running for a long period of time, we set a cutoff time of 4 h. If a test was stopped due to reaching the cutoff time, we replace the ratio by the symbol ‘${{-}{-}}$’, to indicate that the realizability check was aborted after 4 h. For example, in Table 8, for the unrealizable GenBuf specification with 50 senders and up, where a safety guarantee was added for unrealizability, Slugs was stopped after reaching the 4 h cutoff.

In some cases RATSY did not reach the 4 h cutoff, but an ‘Error’ was displayed in the results window in the GUI. This occurred when the NuSMV process fails. We assume it is due to a high memory consumption that we observed at this point. Another failure we experienced is a message ‘unable to allocate X bytes’, which appeared in the ‘Checking outcomes’ window in the GUI, where the NuSMV output is displayed. In the tables, we denote both errors by the symbol ‘$\times \times$’. For example, in Table 8, for the unrealizable GenBuf specification with 60 senders, where a justice guarantee was added for unrealizability, RATSY failed before reaching the 4 h cutoff.

#### Results for AMBA specifications

For both the realizable and the unrealizable AMBA specifications, Spectra completes the realizability check successfully for all sizes (see Table 7). RATSY outperforms Spectra for specifications with at most 4 masters: for the unrealizable specification with 4 masters, where a safety guarantee was added, Spectra is 4 times slower than RATSY. Similarly, Slugs outperforms Spectra for specifications with at most 3 masters. For the realizable specifications Spectra outperforms both tools for specifications with 3 masters and up, and RATSY and Slugs reach the cutoff time for 6 and 5 masters respectively. For unrealizable specifications, Spectra is much faster than in the realizable version, due to the additional heuristic of early unrealizability (see Sect. 3.2.2). Spectra outperforms the other tools for specifications with 5 masters and up. For example, where a justice guarantee was added for unrealizability, Spectra is 500 times faster than both RATSY and Slugs for 6 masters.

For all the specifications, the ratio of the Spectra running time to the other tools decreases as the specifications grow in size.

**Table 8:** Tool comparison over GenBuf specifications

No. of senders	Spectra absolute time	Spectra/RATSY	Spectra/Slugs
5	4.48	38.95	3.46
10	4.87	13.24	7.63
20	6.71	1.12	4.41
30	8.38	0.36	2.06
40	12.73	4.81	0.83
50	20.50	${{-}{-}}$	0.66
60	30.95	$\times \times$	0.64
70	46.21	$\times \times$	0.39
80	67.68	$\times \times$	0.12
90	98.44	$\times \times$	0.30
Realizable specifications			
5	4.46	37.66	22.73
10	4.60	20.86	12.93
20	5.16	4.20	2.19
30	5.86	2.08	1.34
40	6.69	9.13	0.53
50	8.36	${{-}{-}}$	0.32
60	10.46	${{-}{-}}$	0.22
70	12.76	$\times \times$	0.13
80	17.23	$\times \times$	0.08
90	20.67	$\times \times$	0.05
Unrealizable: justice assumption removed			
5	4.44	28.21	1.00
10	4.57	9.34	2.85
20	4.99	0.59	0.29
30	5.50	0.22	0.06
40	6.27	2.24	0.01
50	7.53	${{-}{-}}$	0.002
60	9.10	$\times \times$	0.002
70	10.96	$\times \times$	0.0008
80	13.89	$\times \times$	${{-}{-}}$
90	16.90	$\times \times$	${{-}{-}}$
Unrealizable: additional justice guarantee			
5	4.46	38.89	16.40
10	4.68	1.55	0.41
20	5.30	0.004	0.14
30	6.67	${{-}{-}}$	0.03
40	7.82	${{-}{-}}$	0.01
50	8.93	${{-}{-}}$	${{-}{-}}$
60	11.65	$\times \times$	${{-}{-}}$
70	14.11	$\times \times$	${{-}{-}}$
80	18.78	$\times \times$	${{-}{-}}$
90	22.44	$\times \times$	${{-}{-}}$
Unrealizable: additional safety guarantee			

#### Results for GenBuf specifications

For both the realizable and the unrealizable GenBuf specifications, Spectra completes the realizability check successfully for all sizes (see Table 8). RATSY outperforms Spectra for specifications with at most 40 senders: for the unrealizable specification where a justice assumption was removed, RATSY is 9 times faster than Slugs for 40 senders. However, for specifications with more than 40 senders, RATSY reaches the cutoff time, or encounters a memory problem. Note that the number of assumptions in GenBuf has a polynomial growth in the number of senders (see Appendix A). Since the transition relation is usually the largest BDD with the most expensive computations, we see that RATSY encounters in most specifications memory issues for specifications with 60 senders and up. Slugs, on the other hand, runs successfully and does not reach cutoff time in most cases.

Spectra outperforms Slugs for specifications with 40 senders or more, and the ratio mostly decreases as the specifications grow. For example, where a justice assumption was removed for unrealizability, Spectra is almost 2 times faster than Slugs for specifications with 40 senders and up. For 50 senders Spectra is 3 times faster than Slugs, and it increases with each added number of senders, up to 90 senders, where Spectra is 20 times faster than Slugs.

#### Results summary

The presented results allow us to provide an answer to RQ4: How does the performance of our implementation in Spectra Tools, with all heuristics employed, compare against the performance of previously presented tools?

In realizability checking, the Spectra synthesizer, with all heuristics employed, seems to mostly outperform the previously published tools. For small specifications, RATSY and Slugs are faster than Spectra, but while the other tools cannot complete the realizability check in 4 h, the Spectra tool completes the realizability check in all cases and in many cases, in under a minute. The larger the specification, the faster Spectra becomes relative to the two other tools.

### Threats to validity

We discuss threats to the validity of our results.

#### Internal validity

The implementation of the different heuristics might have bugs, so to ensure correctness of the code we performed validations as described in Sect. 4.3.

Another threat is the variation of the running times of the same test. Different runs of the same algorithm may result in slightly different running times, so the ratios we showed in Sect. 4.4 might not be accurate if we run each test only once. We mitigated this threat by performing 50 runs of each algorithm (or 10 for few very slow executions) and reporting medians as described in Sect. 4.1.

One threat in the comparative tests of the different tools is the different syntax of the specifications. We make sure all tests are executed on the exact same specifications by using the AMBA and GenBuf generators supplied in [13], as well as adding unrealizable options to the generators as described in [13]. The specifications are generated in SMV format, and then translated to all the other tools’ formats. The correctness of the translation scripts was tested with existing specifications in all the formats.

Another threat in the comparative tests is the building of the tools. Both Spectra and Slugs link to the same external library CUDD  [49]. The Spectra tool is written in Java and C (see Sect. 4.1), therefore it links to a single shared object that includes Spectra code and CUDD code. Since the creation of the shared object can affect performance by using different compilation flags, we make sure to use the original build configuration of CUDD in our shared object. As a result, we ensure CUDD’s performance won’t affect the comparative tests.

Finally, another threat to the validity of the comparative tests is the setup of running them. As described in Sect. 4.1, we set up the three tools to use the default dynamic variable reordering of CUDD. We use RATSY with its default settings, and Slugs with the option –fixedPointRecycling. By default, Slugs sets the max memory for the cache in CUDD to 3 GB. For Spectra, the default is 4 GB. All reported ratios are based on tests that run on the same machine and in the same environment. It may be the case that with a different setup, e.g., different dynamic variable reordering choice, different memory settings, etc., results would have been different.

#### External validity

The results of the different heuristics might not be generalizable due to the limited number of specifications used in our evaluation. We divided our evaluation into three sets: (1) SYNTECH15, which are specifications created by students for different robotic systems, (2) SYNTECH17, which are also specifications created by students but with an improved set of tools, and (3) the AMBA and GenBuf specifications, which were created by researchers and systematically scaled to larger sizes. The total number of the specifications might be insufficient. The set SYNTECH15 consists of 78 specifications (17 unrealizable). The set SYNTECH17 consists of 149 specifications (26 unrealizable). The set AMBA and GenBuf consists of 40 specifications (30 unrealizable).

We share some observations on the sets of specifications that might have an influence on the generalizability of the results. First, the AMBA and GenBuf specifications used in the literature were generated systematically for growing parameters (number of AMBA masters and GenBuf senders). Thus the 40 AMBA and GenBuf specifications essentially describe only two systems. Furthermore, the reasons for unrealizability of AMBA and GenBuf were introduced artificially and systematically [13] and consist of a single change each.

Second, the running times for checking realizability of the SYNTECH15 specifications are rather low and range from 1.5 to 1300 ms, with median around 30 ms. In this set the specifications are biased based on the numbers of revisions committed by students during the work on the project: the Humanoid has 21 specifications (8 unrealizable), the Gyro has 11 specifications (2 unrealizable), and the SelfParkingCar has only 4 specifications in total.

Finally, the SYNTECH17 specifications are larger, but the realizability check on 75% of them is still quite fast and takes less than 4 s. The maximal running time is about 8.5 s. There also might be slight bias towards specific sets of specifications: the SIMPLECar has 45 specifications (9 unrealizable), the AirportShuttle has 32 specifications (7 unrealizable), the Elevator has 26 specifications (6 unrealizable), the RobotArm has 26 specifications (2 unrealizable), and the ConvoyCars has 20 specifications (2 unrealizable).

We showed in Sect. 3 that the heuristics’ effect on different specifications can vary considerably. Thus, specifications that share similar characteristics might skew the results in a direction that cannot necessarily be generalized to others. Also, small specifications for which the realizability check is fast to begin with, might not reflect the heuristics in the running times.

We mitigate the above concerns by having on one hand specifications like AMBA and GenBuf that were written by researchers systematically, where we can see the effect of the heuristics on growing specifications, and on the other hand, the SYNTECH15 and SYNTECH17 specifications, which were not created systematically and describe 11 very different Lego robot systems. Also each system has different specifications that are a result of different revisions and they can be very different from one another.

Finally, none of the specifications were written by engineers in an industrial setting, so we cannot evaluate how our results may generalize to large scale real-world specifications in practice.

Despite all the above, it is important to note that in comparison to related works in the field of GR(1) synthesis, the set of specifications that we used for the evaluation is rather large and diverse.

## Related work

We discuss related works that deal with BDD variable reorder and other performance heuristics for reactive synthesis and DDMin.

*BDD variable reorder* It is well known that the order of BDD variables heavily influences the performance of BDD-based algorithms [23, 51]. A lot of work has been done regarding BDD reordering in the context of symbolic representation of states. Ranjan et al. [45] discussed the combination of applying static heuristics to find a good initial order and using dynamic reordering during the computations. They concluded that it is crucial to use dynamic reordering and that the static heuristics have a positive effect. In a more recent work, Kissmann and Hoffmann [26] found that static heuristics are ineffective at reducing BDD size compared to the dynamic reordering.

Our experience showed similar results. Any static reordering heuristics and experiments with employing dynamic reordering at different points did not compare to the dynamic reordering presented by Rudell [46] and implemented in the CUDD package [49] as the default reordering algorithm. The GR(1) implementation of Slugs [17] also uses the default dynamic variable reordering of CUDD, though during strategy construction Slugs turns reordering off. Filippidis et al. [18] reported better performance with reordering during strategy construction. We did not focus on strategy construction in the present work. We are not aware of any GR(1) specific heuristics for dynamic BDD variable ordering. We use CUDD as a black box, therefore improving BDD variable order in the dynamic reordering algorithm itself was beyond the scope of the present work. Still, in our heuristics, we did try to influence reordering from the application side by using grouping of variables with strong affinity, which was suggested in [40], and claimed beneficial for primed and unprimed variables in [51].

*Performance heuristics for reactive synthesis* A number of heuristics for BDD-based safety game solvers have been reported as outcome of the SYNTCOMP reactive synthesis competitions [22–25]. Most of these heuristics are on the level of predecessor computations (operators  in Algorithm 1 and  in Algorithm 2). We employ similar ideas of reordering, eager BDD deallocation, combined conjunction and existential abstraction, and transition partitioning. In addition, we implement heuristics at the level of fixed-points and repeated computations, which were not discussed there. Notably, an approach for predicate abstraction for predecessor computation has already been implemented for GR(1) synthesis [47, 50], and evaluated on a set of specifications for device drivers.

Partitioned transition relation was used in several works [6, 24], though in the context of a simpler case of circuits representations where every latch can be represented by a function. The idea to have smaller BDDs by keeping several parts of a transition relation instead of a single transition was first suggested in [9], and the first automated ordering heuristics for the partitions was given in [20]. This work suggests a greedy algorithm to find the order that will quantify out the most variables in each iteration. A more complex ordering heuristics that uses a cost function to greedily choose the best set of variables to quantify out at each step was suggested in [45].

*Performance heuristics for DDMin* Könighofer et al. [27] presented diagnoses and corresponding algorithms for dealing with unrealizable GR(1) specifications. They also implemented the heuristics for DDMin mentioned in Sect. 3.3.1. They suggest further heuristics that approximate the set of system winning states. These heuristics are different from the ones we presented as they are riskier: in case they fail the computation reverts to the original GR(1) algorithm. An analysis of the speedup obtained from their heuristics for DDMin alone was not reported.

*Strategy construction* Others have focused on strategy construction for GR(1). Strategies are constructed from the memory stored in the X, Y, and Z arrays in Algorithm 1 and Algorithm 2. Schlaipfer et al. [48] suggest synthesis of separate strategies for each justice guarantee to avoid a blow-up of the BDD representation. Bloem et al. [5] discuss minimizations of synthesized strategies that do not necessarily minimize their BDDs. We consider space and time related heuristics for strategy construction an interesting topic for future work.

*Performance heuristics for specifications* Finally, as a very different and complementary approach to ours, one can consider rewriting the GR(1) specification to speed up realizability checking and synthesis. Filippidis et al. [18] report on obtaining a speedup of factor 100 for synthesizing AMBA by manually changing the AMBA specification of [5] to use fewer variables and weaker assumptions. We have not focused on these very specific heuristics of single specifications. Our work presents and evaluates heuristics that are specification agnostic.

## Conclusion

We presented a list of heuristics to potentially reduce running times for GR(1) synthesis and related algorithms. The list includes efficient computations of the controlled predecessors and BDD level operations, specifically grouping of variables and their primed copies, combined conjunction and existential abstraction, and use of partitioned transition relation. It further includes fixed-point related heuristics, specifically early detection of fixed-points, early detection of unrealizability, and fixed-point recycling. Finally, it includes heuristics for accelerating unrealizable core computation.

We have implemented the heuristics and integrated the implementation in the Spectra synthesizer, part of Spectra Tools, a set of tools for writing specifications and running synthesis and related analyses, see [53].

We evaluated the heuristics and their combination on three sets of benchmarks, first SYNTECH15, a set of 78 specifications created by 3rd year undergraduate computer science students in a project class of one semester, second SYNTECH17, a set of 149 specifications created in a repetition of the class, and finally on the two systems, AMBA and GenBuf, which are well-studied in the GR(1) literature. All specifications used in our evaluation, the raw data recorded from all runs, and the program to reproduce our experiments are available from [54].

Our evaluation shows that most heuristics have a positive effect on running times for checking realizability of a specification and for unrealizable core computation. Most importantly, their combination outperforms the individual heuristics, even though there is a risk that some heuristics will increase the running time. It is evident that the greatest improvement is for specifications that have slower original running times. This is a positive result, as these specifications are the ones it is most important to address in order to make GR(1) and related algorithms more useful and applicable in practice.

Moreover, our evaluation shows that the Spectra tool outperforms the two previously published tools, RATSY and Slugs, on large specifications. For small specifications, the overhead of the pre-processing computations is too large compared to the running time of the original implementation. However, the positive effect of the heuristics increases as the specifications grow in size, i.e., the larger the specification, the faster Spectra becomes relative to the two other tools.

We consider the following future research directions. First, one may better investigate why some heuristics do not work well in general and on particular specifications. Perhaps some specifications have special characteristics that make them resist some of the heuristics we have considered. Such an investigation may result in suggestions for additional heuristics, including ones that assist in predicting, given a specification, which if any heuristics may be applied to it successfully.

Second, one may consider the application of the same heuristics to other GR(1) related algorithms, including, e.g., identifying non-well-separation and computing non-well-separation core [34], repair of unrealizable GR(1) specifications [36], etc. These algorithms use building blocks that are very similar to the GR(1) algorithm and thus one may expect similar positive effect of the same heuristics.

Third, we consider further evaluation over additional sets of specifications, both synthetic and real. It may be interesting to evaluate Spectra against the specifications used in the series of SYNTCOMP competitions [22–25]. Note however, that these are either LTL or safety-only specifications. Spectra cannot handle LTL specifications beyond GR(1). While Spectra can handle safety-only specifications, some of our suggested heuristics are irrelevant to these specifications. Our current work, as implemented in Spectra, focuses on GR(1) specifications, which include not only safety but also justice assumptions and guarantees.

Finally, it may be interesting to collect and report not only running time performance data but also iteration depth and memory usage data, specifically comparing the effect of the different heuristics on each of these. Intuitively, we expect the combined conjunction and abstraction heuristics from Sect. 3.1.2 to reduce the maximal memory usage by avoiding the computation of the BDD of the conjunction of the transition relation and the target states. Similarly, we expect the partitioned transition relation heuristics from Sect. 3.1.3 to reduce the maximal memory usage. The fixed-point recycling heuristics from Sect. 3.2.3 is very likely to reduce the number of fixed-point iterations at the cost of additional memory.

Our work is part of a larger project on bridging the gap between the theory and algorithms of reactive synthesis on the one hand and software engineering practice on the other. As part of this project we are developing the Spectra specification language [35] and new algorithms and engineer-friendly tools for reactive synthesis, see, e.g., [1, 30–34, 36].

## Specification sizes

There are three sets of specifications (Sect. 4.2), and each set is divided to realizable and unrealizable specifications. We present in Tables 9 and 10 the sizes of the specifications in these sets in quartiles. Furthermore, we show in Tables 11 and 12 the sizes of the AMBA and GenBuf specifications respectively. All the sizes are rounded to the nearest integer. The sizes are given as follows:

- $2\cdot |\mathcal {X}|$ - the number of primed and unprimed environment variables, equivalent to $|\mathcal {X}\cup \mathcal {X'}|$
- $|\rho ^e|$ - the number of safety assumptions
- $|B_{\rho ^e}|$ - the number of nodes in the BDD of the environment transition relation
- $|J^e|$ - the number of justice assumptions
- $2\cdot |\mathcal {Y}|$ - the number of primed and unprimed system variables, equivalent to $|\mathcal {Y}\cup \mathcal {Y'}|$
- $|\rho ^s|$ - the number of safety guarantees
- $|B_{\rho ^s}|$ - the number of nodes in the BDD of the system transition relation
- $|J^e|$ - the number of justice guarantees

**Table 9:** Realizable specifications’ sizes

Quartile	Assumptions				Guarantees			
	$2\cdot |\mathcal {X}|$	$|\rho ^e|$	$|B_{\rho ^e}|$	$|J^e|$	$2\cdot |\mathcal {Y}|$	$|\rho ^s|$	$|B_{\rho ^s}|$	$|J^s|$
SYNTECH15 realizable								
MIN	2	0	0	0	4	0	4	0
$Q_1$	8	1	7	2	22	18	243	1
$Q_2$	12	2	14	3	40	30	698	2
$Q_3$	20	3	24	4	46	40	1150	2
MAX	26	5	144	7	66	61	4433	4
SYNTECH17 realizable								
MIN	4	0	0	0	0	0	0	0
$Q_1$	15	3	15	0	26	11	687	1
$Q_2$	24	5	91	3	36	16	1499	3
$Q_3$	28	9	758	5	46	38	5551	4
MAX	60	16	80504	19	80	65	88789	6
AMBA/GenBuf realizable								
MIN	10	1	2	2	22	38	199	3
$Q_1$	18	3	6	2	32	65	850	6
$Q_2$	24	11	67	2	44	112	1240	10
$Q_3$	43	42	223	2	61	268	3218	18
MAX	88	87	436	2	106	1005	57668	41

**Table 10:** Unrealizable specifications’ sizes

Quartile	Assumptions				Guarantees			
	$2\cdot |\mathcal {X}|$	$|\rho ^e|$	$|B_{\rho ^e}|$	$|J^e|$	$2\cdot |\mathcal {Y}|$	$|\rho ^s|$	$|B_{\rho ^s}|$	$|J^s|$
SYNTECH15 unrealizable								
MIN	2	0	0	0	10	6	41	0
$Q_1$	6	1	4	2	26	18	276	1
$Q_2$	10	2	14	3	32	27	426	1
$Q_3$	12	2	38	4	46	29	798	2
MAX	26	4	382	5	62	47	3389	4
SYNTECH17 unrealizable								
MIN	12	0	7	0	20	8	657	1
$Q_1$	14	2	17	1	26	16	1130	2
$Q_2$	20	3	41	3	34	23	1703	3
$Q_3$	27	6	167	6	60	36	5197	5
MAX	48	14	11205	18	86	65	31330	6
AMBA/GenBuf unrealizable								
MIN	10	0	0	1	22	38	209	3
$Q_1$	18	3	6	1	30	61	777	6
$Q_2$	24	11	67	2	44	112	1550	10
$Q_3$	48	47	168	2	64	315	3005	21
MAX	88	87	436	2	106	1006	19134	42

**Table 11:** AMBA specifications’ sizes

No. of masters	Assumptions			Guarantees		
	$|\mathcal {X}|$	$|\rho ^e|$	$|J^e|$	$|\mathcal {Y}|$	$|\rho ^s|$	$|J^s|$
1	5	1	2	11	38	3
2	7	2	2	15	60	5
3	9	3	2	19	82	7
4	11	4	2	23	104	9
5	13	5	2	27	126	11
6	15	6	2	30	148	13
7	17	7	2	33	170	15
8	19	8	2	37	192	17
9	21	9	2	41	214	19
10	23	10	2	44	236	21

**Table 12:** GenBuf specifications’ sizes

No. of senders	Assumptions			Guarantees		
	$|\mathcal {X}|$	$|\rho ^e|$	$|J^e|$	$|\mathcal {Y}|$	$|\rho ^s|$	$|J^s|$
5	9	17	2	15	60	6
10	14	27	2	21	120	11
20	24	47	2	32	315	21
30	34	67	2	42	610	31
40	44	87	2	53	1005	41
50	54	107	2	63	1500	51
60	64	127	2	73	2095	61
70	74	147	2	84	2790	71
80	84	167	2	94	3585	81
90	94	187	2	104	4480	91

## Footnotes

1 Following [5], we use the transition relations $\rho ^e$ and $\rho ^s$ inside the LTL formula. Technically, this means prefixing the target states’ variables by the operator **X**.

2 We use Rabin(1) to refer to the dual of GR(1) to avoid confusion with “Generalized Rabin(1) synthesis” as defined by Ehlers [16], where assumptions and guarantees are expressed by generalized Rabin(1) conditions.

3 http://rat.fbk.eu/ratsy/index.php/Main/HomePage.

4 https://github.com/VerifiableRobotics/slugs (commit 1ffbf6b from June 14, 2018).

5 http://smlab.cs.tau.ac.il/syntech/lego/.

6 For the Rabin(1) algorithm (not shown here), this heuristics reduces running times of 25% of the realizable specifications by at most 4% only, and not reduces at all for the unrealizable specifications. This can be attributed to the heuristics details (the difference of GR(1) and Rabin implementations) as described in Sect. 3.1.2.

7 Another observation of Rabin(1) compared to GR(1) is that although the original running times of Rabin(1) are slightly better, with all the heuristics combined the running times are the same or slightly worse in most cases.

8 An interesting observation is that the Rabin(1) algorithm determines realizability faster than the GR(1) algorithm for almost all specifications.

9 We see a greater effect for the Rabin(1) algorithm, especially for specifications for which the original running time is greater than 30. For 25% of these specifications the improvement in running times is at least 25%.

## References

[1] Amram Gal, Maoz Shahar, Pistiner Or (2019). GR(1)*: GR(1) Specifications Extended with Existential Guarantees. Lecture Notes in Computer Science.

[2] Bloem Roderick, Cimatti Alessandro, Greimel Karin, Hofferek Georg, Könighofer Robert, Roveri Marco, Schuppan Viktor, Seeber Richard (2010). RATSY – A New Requirements Analysis Tool with Synthesis. Computer Aided Verification.

[3] Bloem, R., Galler, S.J., Jobstmann, B., Piterman, N., Pnueli, A., Weiglhofer, M.: Interactive presentation: Automatic hardware synthesis from specifications: a case study. In: Lauwereins, R., Madsen, J. (eds.) 2007 Design, Automation and Test in Europe Conference and Exposition, DATE 2007, Nice, France, April 16–20, 2007, pp. 1188–1193. EDA Consortium, San Jose, CA, USA (2007). https://dl.acm.org/citation.cfm?id=1266622

[4] Bloem R, Galler SJ, Jobstmann B, Piterman N, Pnueli A, Weiglhofer M (2007). Specify, compile, run: hardware from PSL. Electr. Notes Theor. Comput. Sci..

[5] Bloem R, Jobstmann B, Piterman N, Pnueli A, Sa’ar Y (2012). Synthesis of reactive(1) designs. J. Comput. Syst. Sci..

[6] Brenguier Romain, Pérez Guillermo A., Raskin Jean-François, Sankur Ocan (2016). Compositional Algorithms for Succinct Safety Games. Electronic Proceedings in Theoretical Computer Science.

[7] Browne A, Clarke EM, Jha S, Long DE, Marrero WR (1997). An improved algorithm for the evaluation of fixpoint expressions. Theor. Comput. Sci..

[8] Bryant RE (1986). Graph-based algorithms for boolean function manipulation. IEEE Trans. Comput..

[9] Burch JR, Clarke EM, Long DE, McMillan KL, Dill DL (1994). Symbolic model checking for sequential circuit verification. IEEE Trans. Comput. Aided Des. Integr. Circuits Sys..

[10] Cavezza Davide G., Alrajeh Dalal (2017). Interpolation-Based GR(1) Assumptions Refinement. Tools and Algorithms for the Construction and Analysis of Systems.

[11] Cerný, P., Kuncak, V., Madhusudan, P. (eds.): Proceedings Fourth Workshop on Synthesis, SYNT 2015, San Francisco, CA, USA, 18th July 2015, EPTCS, vol. 202 (2016). 10.4204/EPTCS.202

[12] Cimatti Alessandro, Clarke Edmund, Giunchiglia Enrico, Giunchiglia Fausto, Pistore Marco, Roveri Marco, Sebastiani Roberto, Tacchella Armando (2002). NuSMV 2: An OpenSource Tool for Symbolic Model Checking. Computer Aided Verification.

[13] Cimatti, A., Roveri, M., Schuppan, V., Tchaltsev, A.: Diagnostic information for realizability. In: VMCAI, LNCS, vol. 4905, pp. 52–67. Springer (2008). 10.1007/978-3-540-78163-9_9

[14] D’Ippolito N, Braberman VA, Piterman N, Uchitel S (2013). Synthesizing nonanomalous event-based controllers for liveness goals. ACM Trans. Softw. Eng. Methodol..

[15] Dwyer, M.B., Avrunin, G.S., Corbett, J.C.: Patterns in property specifications for finite-state verification. In: ICSE, pp. 411–420. ACM (1999). 10.1145/302405.302672

[16] Ehlers Rüdiger (2011). Generalized Rabin(1) Synthesis with Applications to Robust System Synthesis. Lecture Notes in Computer Science.

[17] Ehlers Rüdiger, Raman Vasumathi (2016). Slugs: Extensible GR(1) Synthesis. Computer Aided Verification.

[18] Filippidis Ioannis, Murray Richard M., Holzmann Gerard J. (2016). A multi-paradigm language for reactive synthesis. Electronic Proceedings in Theoretical Computer Science.

[19] Firman Elizabeth, Maoz Shahar, Ringert Jan Oliver (2017). Performance Heuristics for GR(1) Synthesis and Related Algorithms. Electronic Proceedings in Theoretical Computer Science.

[20] Geist Daniel, Beer Ilan (1994). Efficient model checking by automated ordering of transition relation partitions. Computer Aided Verification.

[21] Grädel Erich, Thomas Wolfgang, Wilke Thomas (2002). Automata Logics, and Infinite Games.

[22] Jacobs Swen, Basset Nicolas, Bloem Roderick, Brenguier Romain, Colange Maximilien, Faymonville Peter, Finkbeiner Bernd, Khalimov Ayrat, Klein Felix, Michaud Thibaud, Pérez Guillermo A., Raskin Jean-François, Sankur Ocan, Tentrup Leander (2017). The 4th Reactive Synthesis Competition (SYNTCOMP 2017): Benchmarks, Participants & Results. Electronic Proceedings in Theoretical Computer Science.

[23] Jacobs S, Bloem R, Brenguier R, Ehlers R, Hell T, Könighofer R, Pérez GA, Raskin J, Ryzhyk L, Sankur O, Seidl M, Tentrup L, Walker A (2017). The first reactive synthesis competition (SYNTCOMP 2014). STTT.

[24] Jacobs Swen, Bloem Roderick, Brenguier Romain, Khalimov Ayrat, Klein Felix, Könighofer Robert, Kreber Jens, Legg Alexander, Narodytska Nina, Pérez Guillermo A., Raskin Jean-François, Ryzhyk Leonid, Sankur Ocan, Seidl Martina, Tentrup Leander, Walker Adam (2016). The 3rd Reactive Synthesis Competition (SYNTCOMP 2016): Benchmarks, Participants & Results. Electronic Proceedings in Theoretical Computer Science.

[25] Jacobs Swen, Bloem Roderick, Brenguier Romain, Könighofer Robert, Pérez Guillermo A., Raskin Jean-François, Ryzhyk Leonid, Sankur Ocan, Seidl Martina, Tentrup Leander, Walker Adam (2016). The Second Reactive Synthesis Competition (SYNTCOMP 2015). Electronic Proceedings in Theoretical Computer Science.

[26] Kissmann P, Hoffmann J (2014). BDD ordering heuristics for classical planning. J. Artif. Int. Res..

[27] Könighofer R, Hofferek G, Bloem R (2013). Debugging formal specifications: a practical approach using model-based diagnosis and counterstrategies. STTT.

[28] Kozen D (1983). Results on the propositional mu-calculus. Theor. Comput. Sci..

[29] Kress-Gazit H, Fainekos GE, Pappas GJ (2009). Temporal-logic-based reactive mission and motion planning. IEEE Trans. Robot..

[30] Kuvent, A., Maoz, S., Ringert, J.O.: A symbolic justice violations transition system for unrealizable GR(1) specifications. In: Bodden, E., Schäfer, W., van Deursen, A., Zisman, A. (eds.) Proceedings of the 2017 11th Joint Meeting on Foundations of Software Engineering, ESEC/FSE 2017, Paderborn, Germany, September 4–8, 2017, pp. 362–372. ACM (2017). 10.1145/3106237.3106240

[31] Maoz Shahar, Pistiner Or, Ringert Jan Oliver (2016). Symbolic BDD and ADD Algorithms for Energy Games. Electronic Proceedings in Theoretical Computer Science.

[32] Maoz, S., Ringert, J.O.: GR(1) synthesis for LTL specification patterns. In: Nitto, E.D., Harman, M., Heymans, P. (eds.) Proceedings of the 2015 10th Joint Meeting on Foundations of Software Engineering, ESEC/FSE 2015, Bergamo, Italy, August 30–September 4, 2015, pp. 96–106. ACM (2015). 10.1145/2786805.2786824

[33] Maoz Shahar, Ringert Jan Oliver (2016). Synthesizing a Lego Forklift Controller in GR(1): A Case Study. Electronic Proceedings in Theoretical Computer Science.

[34] Maoz, S., Ringert, J.O.: On well-separation of GR(1) specifications. In: Zimmermann, T., Cleland-Huang, J., Su, Z. (eds.) Proceedings of the 24th ACM SIGSOFT International Symposium on Foundations of Software Engineering, FSE 2016, Seattle, WA, USA, November 13–18, 2016, pp. 362–372. ACM (2016). 10.1145/2950290.2950300

[35] Maoz, S., Ringert, J.O.: Spectra: a specification language for reactive systems. arXiv:1904.06668 (2019)

[36] Maoz, S., Ringert, J.O., Shalom, R.: Symbolic repairs for GR(1) specifications. In: Mussbacher, G., Atlee, J.M., Bultan, T. (eds.) Proceedings of the 41st International Conference on Software Engineering, ICSE 2019, Montreal, QC, Canada, May 25–31, 2019, pp. 1016–1026. IEEE/ACM (2019). https://dl.acm.org/citation.cfm?id=3339632

[37] Maoz, S., Sa’ar, Y.: AspectLTL: an aspect language for LTL specifications. In: Borba, P., Chiba, S. (eds.) AOSD, pp. 19–30. ACM (2011). 10.1145/1960275.1960280

[38] Maoz Shahar, Sa’ar Yaniv (2012). Assume-Guarantee Scenarios: Semantics and Synthesis. Model Driven Engineering Languages and Systems.

[39] Maoz S, Sa’ar Y (2013). Two-way traceability and conflict debugging for aspectltl programs. Trans. Aspect Oriented Softw. Dev..

[40] Panda, S., Somenzi, F.: Who are the variables in your neighborhood. In: Proceedings of the 1995 IEEE/ACM International Conference on Computer-aided Design, ICCAD ’95, pp. 74–77. IEEE Computer Society, Washington, DC, USA (1995). http://dl.acm.org/citation.cfm?id=224841.224862

[41] Piskac, R., Dimitrova, R. (eds.): Proceedings Fifth Workshop on Synthesis, SYNT at CAV 2016, Toronto, Canada, July 17–18, 2016, EPTCS, vol. 229 (2016). 10.4204/EPTCS.229

[42] Piterman Nir, Pnueli Amir, Sa’ar Yaniv (2005). Synthesis of Reactive(1) Designs. Lecture Notes in Computer Science.

[43] Pnueli, A., Rosner, R.: On the synthesis of a reactive module. In: POPL, pp. 179–190. ACM Press (1989). 10.1145/75277.75293

[44] Pnueli Amir, Sa’ar Yaniv, Zuck Lenore D. (2010). Jtlv: A Framework for Developing Verification Algorithms. Computer Aided Verification.

[45] Ranjan, R.K., Aziz, A., Brayton, R.K., Plessier, B., Pixley, C.: Efficient BDD algorithms for FSM synthesis and verification. In: In IEEE/ACM Proceedings International Workshop on Logic Synthesis, Lake Tahoe, NV (1995)

[46] Rudell R. (2003). Dynamic Variable Ordering for Ordered Binary Decision Diagrams. The Best of ICCAD.

[47] Ryzhyk Leonid, Walker Adam (2016). Developing a Practical Reactive Synthesis Tool: Experience and Lessons Learned. Electronic Proceedings in Theoretical Computer Science.

[48] Schlaipfer Matthias, Hofferek Georg, Bloem Roderick (2012). Generalized Reactivity(1) Synthesis without a Monolithic Strategy. Hardware and Software: Verification and Testing.

[49] Somenzi, F.: CUDD: BDD package, University of Colorado, Boulder. http://vlsi.colorado.edu/~fabio/CUDD/cudd.pdf

[50] Walker, A., Ryzhyk, L.: Predicate abstraction for reactive synthesis. In: Formal Methods in Computer-Aided Design, FMCAD 2014, Lausanne, Switzerland, October 21–24, 2014, pp. 219–226. IEEE (2014). 10.1109/FMCAD.2014.6987617

[51] Yang B, Bryant RE, O’Hallaron DR, Biere A, Coudert O, Janssen G, Ranjan RK, Somenzi F, Gopalakrishnan G, Windley P (1998). A performance study of BDD-based model checking. Formal Methods in Computer-Aided Design.

[52] Zeller A, Hildebrandt R (2002). Simplifying and isolating failure-inducing input. IEEE Trans. Software Eng..

[53] Spectra Website. http://smlab.cs.tau.ac.il/syntech/spectra/

[54] SYNTECH GR(1) Performance Website. http://smlab.cs.tau.ac.il/syntech/performance/

